# Supramolecular janus eutectic engineered from panthenol and bisabolol overcomes transdermal delivery paradoxes

**DOI:** 10.1038/s41467-026-77613-x

**Published:** 2026-09-08

**Authors:** Hao WANG, Qingsheng TAO, Xiaofeng HE, Kung Chi HSU, Yu GAO, Delphine KEROB, Wei LIU, Zhenyuan WANG, Nan HUANG, Jiaheng ZHANG

**Affiliations:** 1 Sauvage Laboratory for Smart Materials, School of Materials Science and Engineering, Harbin Institute of Technology (Shenzhen), Shenzhen, China; 2 L’Oreal Research & Innovation China, Shanghai, China; 3 La Roche-Posay Laboratoire Dermatologique, La Roche-Posay, Levallois-Perret, France; 4 Department of Dermatology, The General Hospital of Air Force PLA, Beijing, China; 5 Shenzhen Shinehigh Innovation Co., Ltd, Shenzhen, China

**Keywords:** Drug delivery, Biotechnology, Pharmacology

## Abstract

Conventional transdermal delivery systems are constrained by inherent trade-offs among penetration efficiency, bioactive potency, and skin biocompatibility. To overcome these limitations, here we show a supramolecular Janus eutectic engineered from D-panthenol and α-(-)-bisabolol (SPB). This carrier-free system effectively integrates autonomous permeation with multimodal therapeutic functions. SPB demonstrates synergistic enhancement of panthenol transdermal delivery while markedly improving the biocompatibility of bisabolol, alongside multiplex anti-irritation and pro-repair efficacy—a combination unattainable by its individual components or physical mixtures. Mechanistic investigations indicate that the supramolecular architecture modulates the active transport pathway and antagonizes the TRPV1 receptor. This work provides a practical strategy for designing biofunctional materials that intrinsically unify delivery, efficacy, and safety.

## Abstract (web summary)

Transdermal delivery systems are constrained by a trade off in penetration efficiency bioactivity. Here, the authors report on A supramolecular eutectic of D-panthenol and bisabolol that overcomes the trade-offs, achieving synergistic transdermal efficacy and skin repair that physical mixtures cannot replicate

## Introduction

The long-term management of dermatological conditions with underlying inflammation (e.g., sensitive skin, eczema, acute wounds) presents a fundamental therapeutic paradox: the intrinsic conflict between achieving high topical pharmacological efficacy and maintaining skin barrier integrity. While potent anti-inflammatory effects are desirable, with corticosteroids being the cornerstone for acute phase management^1,2^, their long-term effect on skin recovery and barrier function could introduce unwanted outcomes such as slowed wound healing and higher susceptibility to irritation. In clinical practice, the mainstream approach has been using corticosteroids to contain acute phase inflammation with intense-dose short duration therapy, in order to avoid unwanted collateral effects such as epidermal atrophy and impaired barrier recovery. This efficacy-safety-barrier trilemma has remained an unresolved challenge in materials science, medicine, and interdisciplinary research, calling for innovative strategies that simultaneously optimize bioavailability, biocompatibility, and therapeutic outcomes.

Current transdermal delivery systems have failed to resolve this paradox. Chemical enhancers^3^ (e.g., ethanol, ozone) and physical approaches (e.g., microneedles^4^) achieve drug delivery efficacy at the expense of barrier disruption, while nanocarriers (liposomes^5^, polymeric micelles^6^) face limitations in manufacturing complexity^7^, carrier toxicity^8^, and stability^9^. Although emerging ionic liquid-based delivery systems^10^ show promise, they are constrained by limited ligand options for irritation mitigation and immature industrial production standards^11^. These technologies universally treat carrier and therapy as separate entities, overlooking the innate delivery potential of bioactive molecules themselves. Deep Eutectic Solvents (DES) are characterized by a melting point significantly lower than that of any individual component^12^. As most components are solids with similar physicochemical properties, high viscosity is also a notable feature of DES, which constitutes a core bottleneck limiting its full functional potential and industrial application. Nevertheless, the good biocompatibility and synergistic functions of DES still offer considerable application value, with Natural Deep Eutectic Solvents (NaDES) being particularly favored^13^. Based on DES, we propose the concept of JANUS Eutectics, emphasizing that the precursors are composed of components with substantially different or even opposite properties (e.g., hydrophilic vs. lipophilic) yet functionally complementary. The supramolecular structure is maintained by hydrogen bonding, with the glass transition temperature (Tg) as the core characteristic, and exhibits the following properties: (1) bioactive components serve as building blocks with unified yet complementary properties, (2) inherent integration of delivery and therapeutic functions, and (3) resolution of the historical efficacy-barrier-irritation paradox.

Provitamin B5, also known as D-panthenol, is one of the most used provitamins in topical skin repair^14^, burn/ulcer treatment^15^, ophthalmology^16^, and hair care^17^ formulations, responsible for enhancing wound healing via promoting cellular metabolism and cell proliferation^18,19^. The logarithm of the partition coefficient (Log P) is a quantitative measure of compound lipophilicity and can be calculated using various models^20^, such as SwissADME. Log P is commonly used to preliminarily assess the ease with which an active agent penetrates the skin lipid barrier. It exhibits an inverted U-shaped parabolic relationship with permeation efficiency (Hansch model), with the optimal range being 1–4. D-Panthenol’s polyhydroxy structure confers strong hydrophilicity (log *P* ≈ −0.8), which promotes preferential partitioning into the *stratum corneum*’s aqueous boundary layer rather than transcellular penetration. This results in significant corneocyte retention but limited deeper permeation^21^, creating a classic hydrophilic permeation paradox^22^. α-(-)-Bisabolol is an unsaturated, optically active sesquiterpene alcohol found as a major component of the essential oil of chamomile. Both chamomile extracts and α-(-)-bisabolol are widely incorporated into topical formulations for oral mucositis^23^, gingivitis^24^, dermatitis^25^, and acne vulgaris, with their well-known anti-inflammatory potential^26^. Notably, α-(-)-bisabolol exhibits good skin penetration performance due to its lipophilic properties (log *P* = 4.2), which facilitate partitioning into intercellular lipids of the *stratum corneum*^27,28^. Ethoxydiglycol, with a trade name of Transcutol®, is a liquid that has a long history of use in cosmetic and over-the-counter topically applied products and is generally considered as safe^29^. Ethoxydiglycol ether is also very attractive as a penetration enhancer, and its presence could further improve the skin penetration of the supramolecular system^30^.

Through proton exchange reactions, we synthesized a supramolecular D-panthenol-ɑ-(-)-bisabolol Janus eutectic (SPB), with ethoxydiglycol employed to ensure structural stability and prevent phase separation. Our multiscale investigation spanning quantum chemical calculations based on Density Functional Theory (DFT)^31^, molecular dynamics simulations^32^, and organoid models^33^ - elucidates three fundamental scientific principles of SPB: (1) Bioavailability enhancement mechanism: Optimized electrostatic potential distribution enabling amphiphilicity; Decreased intercellular lipid pathway resistance; Lower energy consumption during permeation. (2) Biocompatibility preservation mechanism: Enhanced paracellular delivery efficiency; Supramolecular shielding of reactive functional groups; Self-limiting interaction with *stratum corneum* components. (3) Synergistic therapeutic mechanism: Superior binding affinity to the transient receptor potential cation channel subfamily V member 1 (TRPV1) and modulation of downstream calcium influx pathways to mitigate nociception instead of tampering with the entire immune homeostasis.

This work provides a proof-of-concept for carrier-free delivery systems and offers a promising approach for reconstructing bioactive natural molecules through supramolecular engineering. By elucidating a previously underexplored aspect of the interaction between active ingredients and delivery systems, this work provides a practical design strategy for advancing dermatological therapeutics.

## Results and discussion

### Synthesis and Properties of SPB

**Fig. 1:**
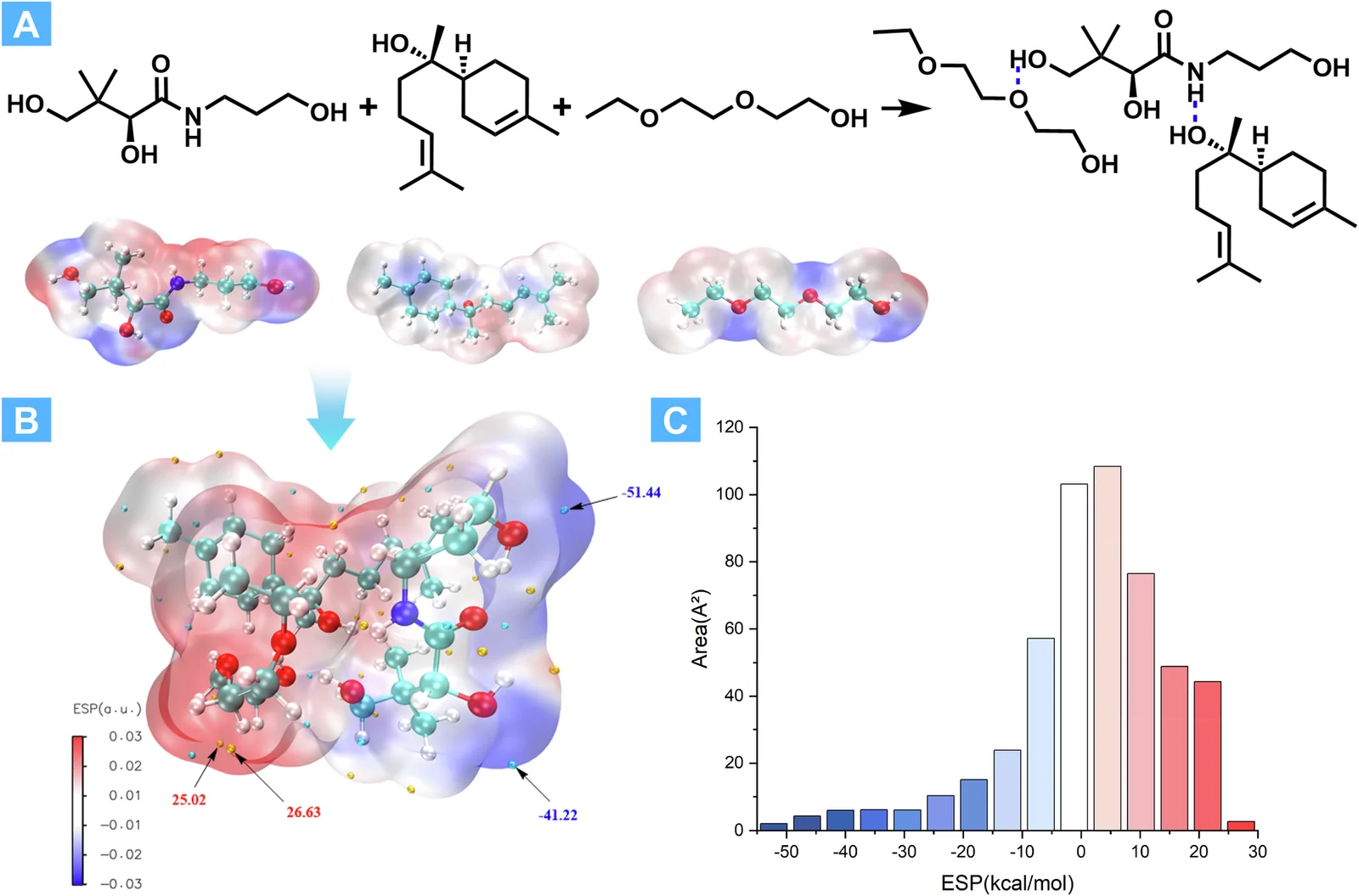
SPB Synthesis and ESP Changes. **A** Synthetic route of SPB. **B** ESP of SPB. **C** Area distribution of ESP for SPB.

SPB was prepared by mixing designed amounts of D-Panthenol, α-(-)-bisabolol, and ethoxydiglycol at a molar ratio of 1:1:1 in a vacuum. SPB was generated by hydrogen bonding interactions rather than covalent bonds. Hydrogen bonding interaction happened between D-Panthenol and ɑ-(-)-bisabolol, partially existed between D-Panthenol and ethoxydiglycol, as shown in Fig. 1A. The Independent Gradient Model based on Hirshfeld partition (IGMH)^34^ effectively elucidates the intermolecular interactions within SPB. It can be clearly observed that the amino group of D-panthenol and the hydroxyl group of ɑ-(-)-bisabolol form strong hydrogen bonds, while the ether bond near the hydroxyl group of ethoxydiglycol forms hydrogen bonds with the hydroxyl group of D-panthenol. Interactions at other sites are predominantly van der Waals forces, corresponding to Fig. 1A.

**Fig. 2:**
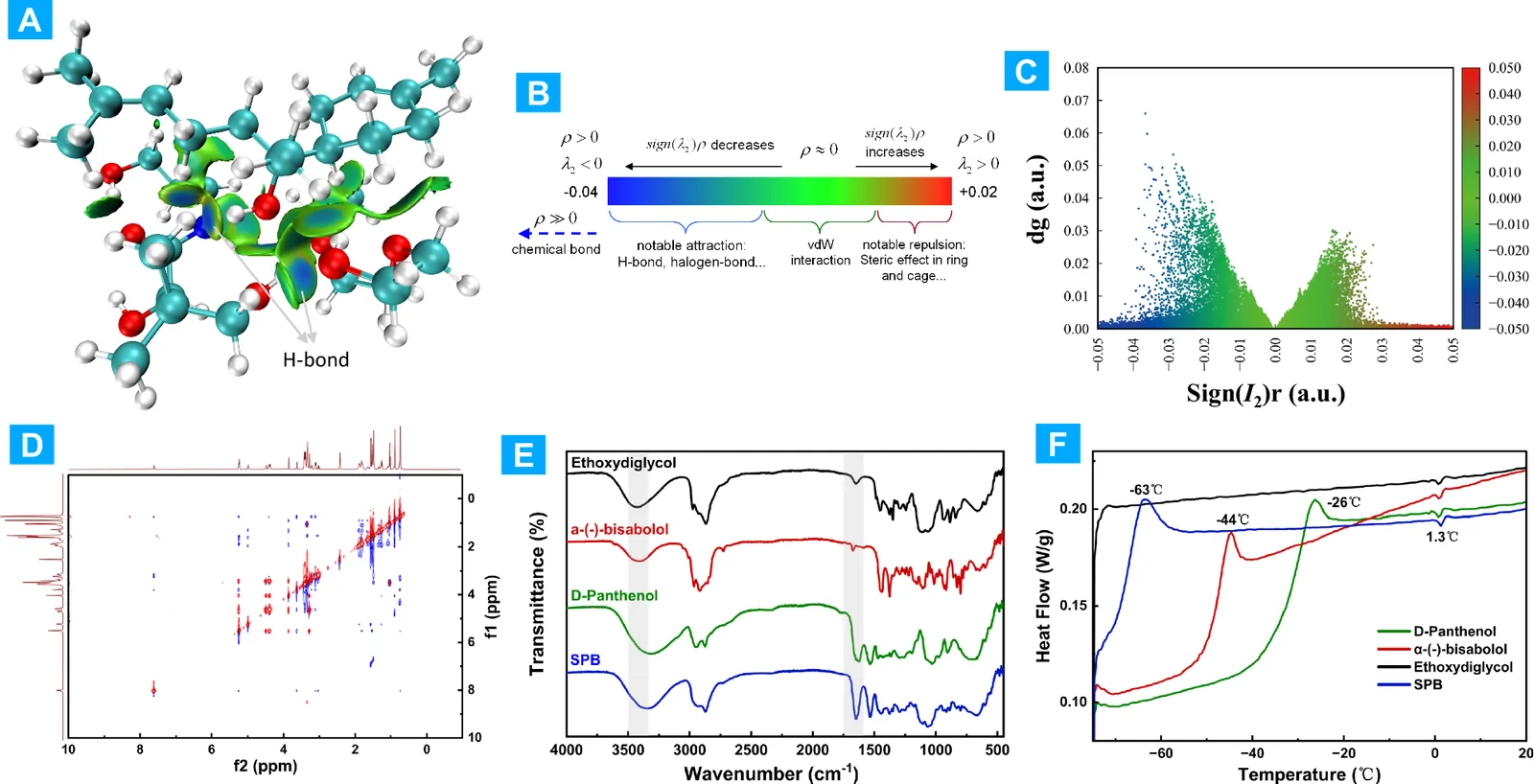
Structural and characterization of SPB. **A** IGMH visualization of SPB. The turquoise isosurfaces represent intermolecular interaction regions, with color mapping indicating interaction types and strengths. **B** Interaction type color scheme. **C** Scatter plot of IGMH electron density distribution. **D** NOSEY of SPB. **E** FTIR of SPB and individual components. **F** DSC and Tg of SPB and individual components.

The isosurfaces delineate interaction zones (Fig. 2A), with color mapping indicating interaction types and strengths (Fig. 2B): blue regions represent hydrogen-bonding attractions, while red denotes repulsive forces. Enhanced electron density in interaction zones correlates with stronger atomic interactions, evidenced by higher peak intensities in the scatter plot (Fig. 2C), which correspond to shorter interatomic distances and constancy of tight integration. Comparative analysis reveals that D-panthenol, α-(-)-bisabolol, and ethoxydiglycol formed stable supramolecular Janus units through hydrogen bonding and other non-covalent attractions, with negligible repulsive interactions–a key factor underlying the system’s homogeneous stability. This theory has been validated in H nuclear magnetic resonance spectra (¹H NMR), nuclear overhauser effect spectroscopy (NOESY), Fourier-transform infrared spectroscopy (FTIR), and differential scanning calorimetry (DSC). ¹H NMR exhibits the composition of functional groups and the analogous peak shifts caused by hydrogen bonding effects (Supplementary Fig. 1). Two-dimensional NMR further visualizes hydrogen bond locations through cross-peaks arising from hydrogen atom coupling. The NOESY spectrum (Fig. 2D) shows distinct cross-peaks at 7.7 ppm (D-panthenol amide) and 0.6-5.3 ppm (hydroxyl groups), precisely matching the predicted hydrogen bond positions. The consistent results are also presented in FTIR (Fig. 2E): SPB’s structure relies on hydrogen bonding between D-panthenol’s amide group (showing a single peak at 3500–3050 cm^−1^ and a free-state characteristic peak at 1600 cm^−1^) and the hydroxyl groups of ɑ-(-)-bisabolol/ethoxydiglycol (broad single peak at 3700–3200 cm^−1^ with trough at 3450 cm^−1^). Hydrogen bonding induces characteristic red shifts: the hydroxyl trough shifts from 3450–3350 cm^−1^, while the amide peak moves from 1600–1640 cm^−1^. Significant modifications also occur in the alcohol hydroxyl region (1200–1000 cm^−1^). The extensive intramolecular hydrogen bonding network restricts molecular mobility, enabling differentiation between supramolecular and free states through glass transition temperature (Tg) analysis. The DSC thermogram (Fig. 2F) clearly demonstrates a significant decrease in Tg, which is an important indicator of the successful synthesis of SPB. These collective findings verify SPB’s unique supramolecular architecture and its consequent impact on material characteristics. For example, electrostatic potential (ESP)^35^ analysis reveals localized charge distribution and polarity, caused by intermolecular interactions. Figure 1B illustrates the ESP distribution of SPB, revealing significant charge redistribution under hydrogen bonding interactions. This reorganization results in expanded areas of concentrated like charges, which may positively influence skin affinity and permeation enhancement. The interval distribution of ESP extremum points (Fig. 1C) demonstrates numerous negative charge maxima, while neutral charge regions occupy the largest surface area–a characteristic that crucially determines SPB’s amphiphilicity and potential permeation behavior in skin tissue^36,37^.

Stress tests (Supplementary Information) demonstrate that SPB maintains structural integrity under harsh conditions, including a wide pH range (3–11, Supplementary Table 2 and Supplementary Fig. 2), extreme temperatures (Supplementary Table 3), and exposure to strong oxidizing agents (e.g., H_2_O_2_, Supplementary Table 4). Current knowledge gaps: The working mechanism and mechanistic principles governing supramolecular systems remain insufficiently systematized, impeding their cross-disciplinary applications.

### Bioavailability enhancement mechanism of SPB

**Fig. 3:**
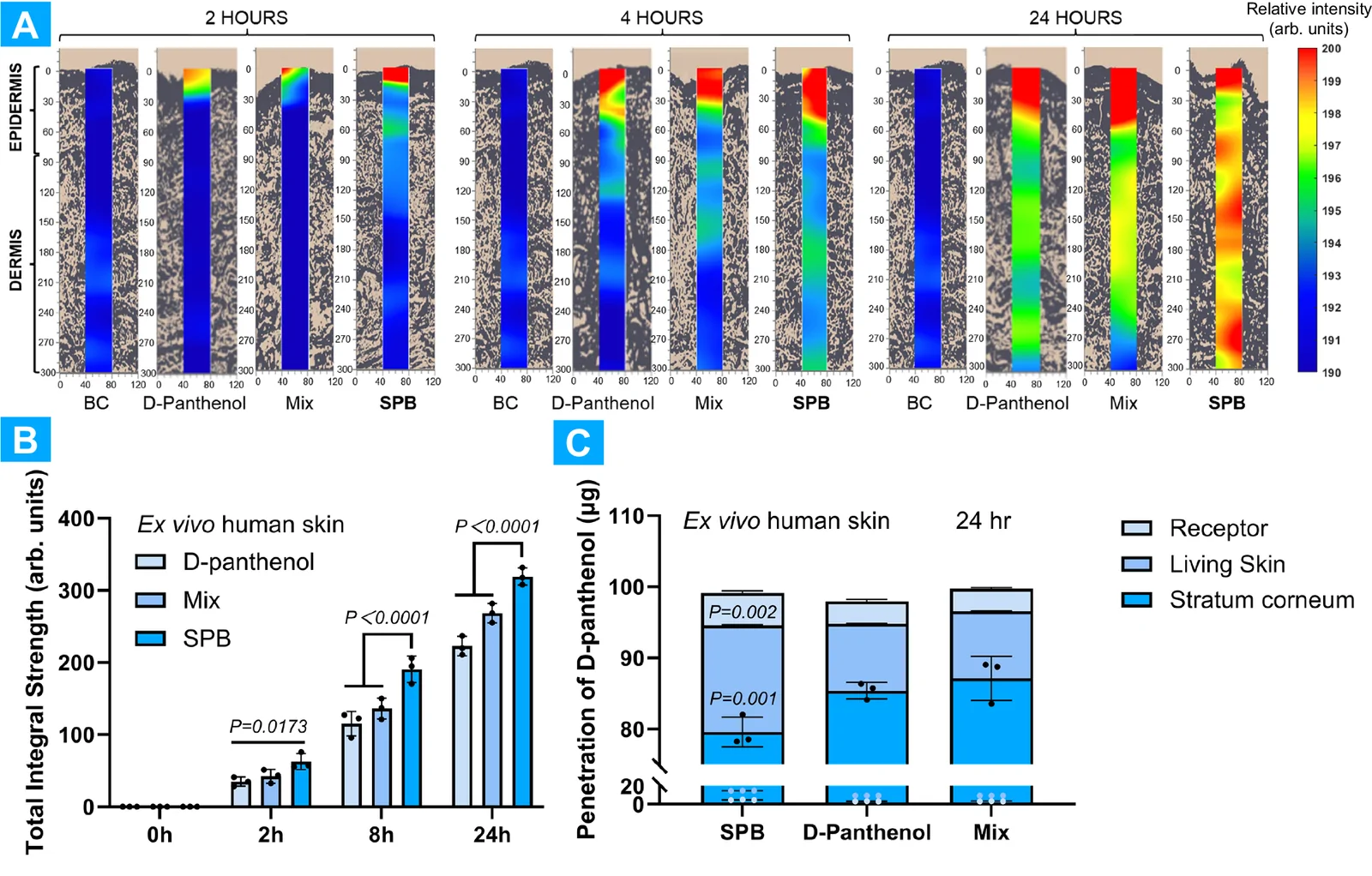
Analysis of bioavailability of D-panthenol in various formats. **A** Pseudo-color image of transdermal penetration at different time points (representative image from 3 independent experiments is shown; similar results were obtained in all 3 independent repeats). **B** Histogram of total integral strength (1301 cm^−1^). Results are shown as mean ± SD. *N* = 3 skin explants per group (the unit of study is the individual skin explant, each obtained from a different donor; the same donor was not used for multiple explants). Within each experiment, technical triplicates (three measurements per group) were averaged before statistical analysis. Two-way ANOVA followed by Tukey’s multiple comparison test, two-sided. Groups compared: SPB *vs*. D-panthenol in 2 h: Mean difference = −27.97, 95% CI [−51.42, −4.519], q = 4.212, F = 24, adjusted *P* = 0.0173. **C** Quantitative penetration of D-panthenol in various formats. Results are shown as mean ± SD. *N* = 3 skin explants per group, and then technical triplicates (three measurements per sample) for each experiment. Two-way ANOVA followed by Tukey’s multiple comparison test, two-sided. Groups compared: SPB *vs*. D-panthenol: Mean difference = −5.800, 95% CI [−8.540, −3.060], q = 7.639, adjusted *P* = 0.0001; Mean difference = 5.833, 95% CI [2.793, 8.274], q = 7.288, F = 18, adjusted *P* = 0.0002.

To improve translational relevance, this study utilized ex vivo human skin rather than animal experimentation. With a standard Franz-cell diffusion cell penetration test, the full-layer multi-point Raman scanning spectrum of the skin was acquired at various permeation time points using a confocal Raman spectrometer. The pseudo-color image reconstruction could be performed with the Witec data analysis software by using the comprehensive intensity of the characteristic peak at 1301 cm^−1^, as it differs from endogenous skin Raman signals and exhibits strong detectability even at low concentrations. The intensity of the characteristic peak was related to the brightness of the Raman image color (Fig. 3A), and semi-quantitatively analyzed the characteristic peaks of the substance (Fig. 3B). At the concentration of 0.3%, the total integral intensity under the 1301 cm^−1^ peak of the sample SPB increased significantly after 2 h, 8 h and 24 h, compared with panthenol at the same concentration, and the improvement rates were 80.93%, 65.57% and 43.36%, respectively. Compared with the simple mixture group (Mix), the improvement rates were 48.17%, 39.91% and 18.91%, respectively. Furthermore, Fig. 3A visually demonstrates the superior penetration depth and efficiency of SPB at all time points. This highlights a clear distinction in transdermal permeation between the supramolecular SPB and a simple mixture of equivalent content, even within a minimal supply chamber under heated and agitated conditions. HPLC quantification method of D-panthenol was confirmed to be reliable as shown in Supplementary Fig. 3, with standard curve linearity reaching *R*^2^ ≥ 0.995 and lowest quantification limit at 0.195 μg/mL. As indicated in Fig. 3C, at the end of the 24 h incubation time, SPB was able to penetrate through skin tissue towards receptor fluid to a greater extent in comparison to other formats with the same level of D-panthenol (46% and 44% higher, respectively, *P* < 0.01). There was significantly less D-panthenol on the skin surface for the supramolecular format, corresponding to a higher amount penetrated into the skin (living skin and receptor), and the overall bioavailability increased by 55.4% and 54.8%, respectively (*P* < 0.01). At the same time, there was no apparent difference among the treatments regarding recovery rate for the total applied dosage. The above-mentioned results suggested that the supramolecular structure altered the molecular dynamics underlying transdermal penetration, leading to enhanced bioavailability of panthenol, without the introduction of conventional penetration enhancers such as sodium lauryl sulfate (SLS) and Tween, or mechanical enhancement, e.g., ultrasound^38,39^. This observation is in line with previously reported delivery-promoting effects associated with supramolecules, through modification of biocompatibility, stability, and spatiotemporal distribution^40^.

**Fig. 4:**
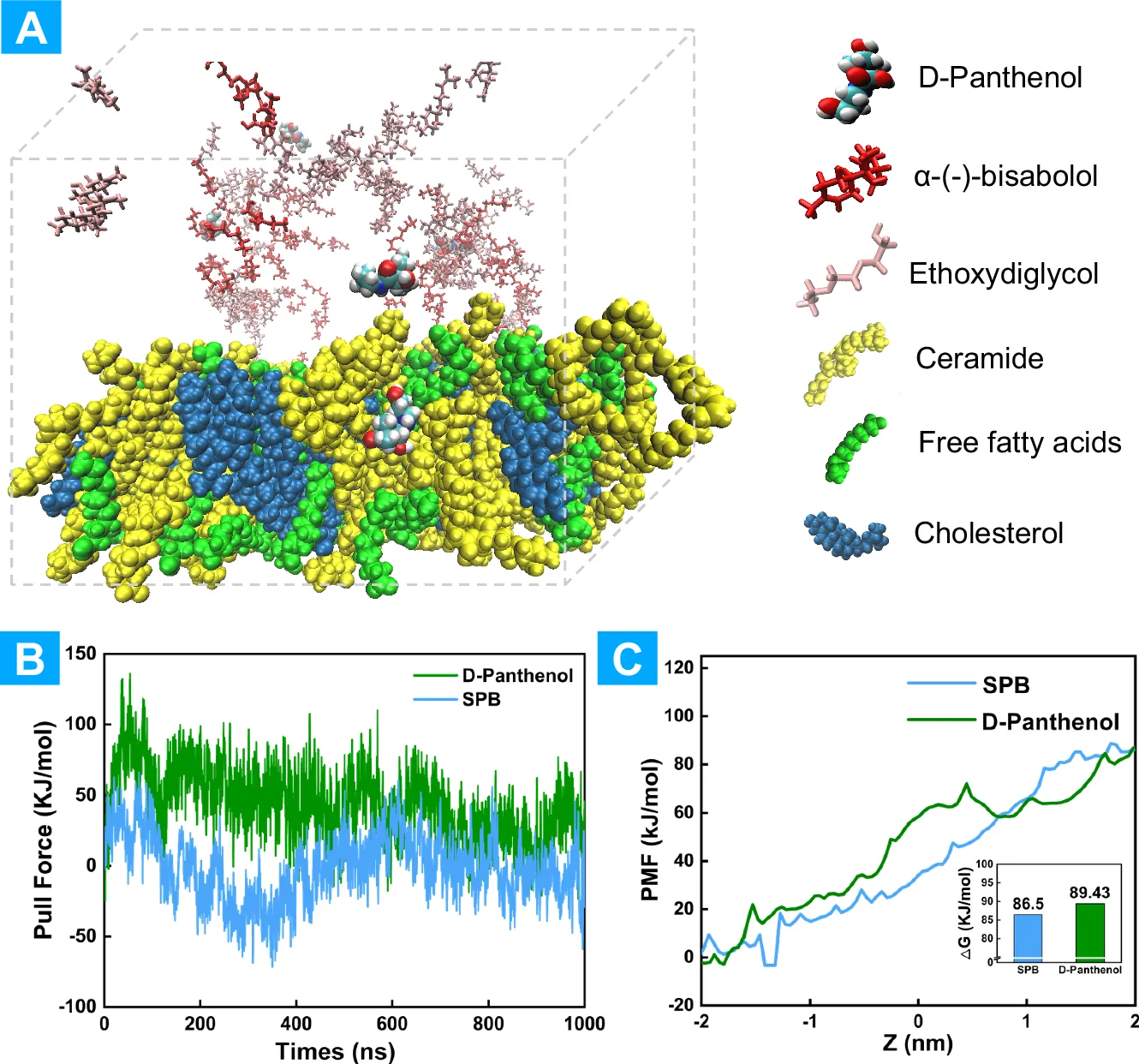
Molecular dynamics simulation of the SPB transmembrane process. **A** Lipid Bilayer Permeation Model. **B** Resistance in transmembrane transport. **C** PMF (potential of mean force) profiles and Energy expenditure.

To further explore the molecular interactions between different forms of D-panthenol and the *stratum corneum* (SC), an in silico simulation was conducted, with a simplified SC lipid bilayer comprised of ceramide (CER), cholesterol (CHOL), and free fatty acids (FFA) in a 1:1:1 molar ratio^41^. Figure 4A provides direct visualization of the transmembrane processes for SPB across the lipid bilayer, with Supplementary Fig. 4 (supplementary video) further demonstrating the dynamic interactions during SPB permeation. The simulation system maintained excellent stability throughout the transmembrane process without any structural anomalies, as confirmed by the molecular density profile (Supplementary Fig. 5), which validates the reliability of our molecular dynamics model. Comparative analysis of transmembrane resistance (Fig. 4B) reveals significantly reduced resistance for SPB compared to free D-panthenol, likely attributable to SPB’s amphiphilic. The potential of mean force (PMF) profiles (Fig. 4C) quantitatively describe the energy landscape during permeation, with −2 nm and 2 nm representing initial contact and complete crossing of the lipid model, respectively.

Molecular dynamics analysis reveals that upon contact with the lipid bilayer, D-panthenol exhibits negative potential of PMF values due to its inherent hydrophilicity and strong affinity for the hydrophilic headgroups of the bilayer. In contrast, during separation from the bilayer, SPB demonstrates greater PMF expenditure than D-panthenol, attributed to its enhanced lipid affinity conferred by the supramolecular architecture. The energy difference between these points demonstrates that SPB requires substantially less energy to overcome the lipid barrier^42^. Compared with the single‑component ESP (Supplementary Figs. 6–8), SPB exhibits more concentrated polar and non‑polar surfaces, significantly enhancing its amphiphilicity. Furthermore, SPB has a log P of 1.32, which lies within the ideal transdermal range (1‑4). These collective findings support that SPB’s superior bioavailability stems from its optimized transmembrane behavior, providing a molecular-level foundation for its enhanced therapeutic efficacy.

### Biocompatibility and skin-repairing potential of SPB

**Fig. 5:**
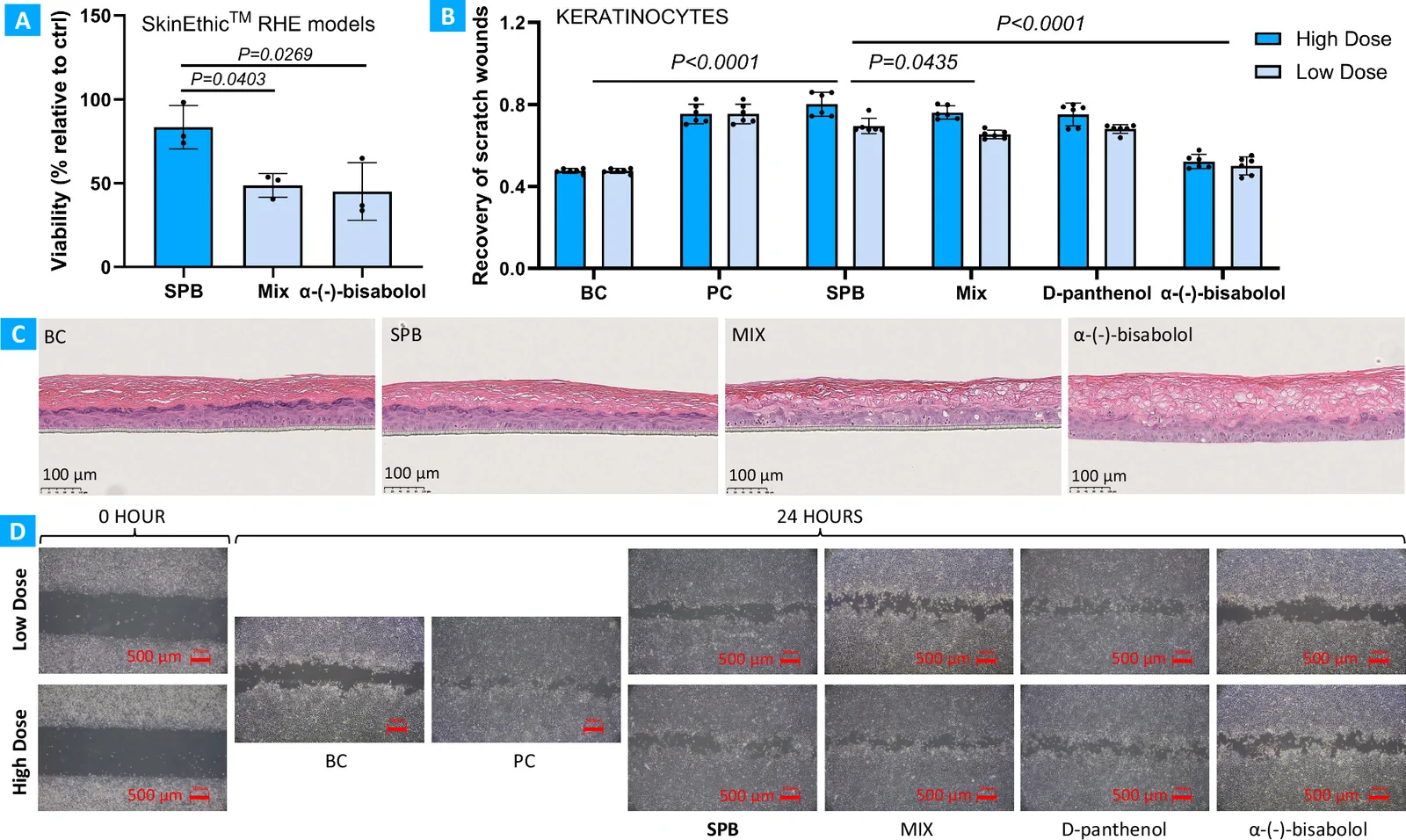
Biocompatibility and repair potential evaluation of SPB. **A** Viability of RHE models treated with the actives. Results are shown as mean ± SD. *N* = 3 biologically independent per group (each experiment used primary keratinocytes isolated from a different donor; the unit of study is the individual isolation). Within each experiment, technical triplicates (three wells per condition) were averaged before statistical analysis. One-way ANOVA followed by Tukey’s multiple comparison test, two-sided. Groups compared: SPB *vs*. Mix: Mean difference = 34.73, 95% CI [1.893, 67.57], q = 4.589, F = 6, adjusted *P* = 0.0403. SPB *vs*. α-(-)-bisabolol: Mean difference = 38.37, 95% CI [5.526, 71.21], q = 5.069, F = 6, adjusted *P* = 0.0269. **B** Keratinocyte wound healing potential of bioactive molecules. Detailed dosages are shown in Supplementary Table 5. Recovery area (% of total scratched area relative to blank control at baseline) shown as mean ± SD. *N* = 3 biologically independent per group (each experiment used primary keratinocytes isolated from a different donor; the unit of study is the individual isolation), and then technical triplicates for each experiment (three wells per condition). Two-way ANOVA followed by Dunnett’s multiple comparison test, two-sided. Groups compared: SPB *vs*. BC: Mean difference = 0.2726, 95% CI [0.2320, 0.3132], q = 4.589, F = 60, adjusted *P* < 0.0001. SPB *vs*. Mix: Mean difference = 0.04147, 95% CI [0.0008925, 0.08205], q = 2.639, F = 60, adjusted *P* = 0.0435. SPB *vs*. α-(-)-bisabolol: Mean difference = 0.2376, 95% CI [0.1971, 0.2782], q = 15.12, F = 60, adjusted *P* < 0.0001. **C** H&E histological morphology (representative image from 3 independent experiments is shown; similar results were obtained in all 3 independent repeats) of RHE models treated with different actives. **D** Image of keratinocyte wound healing after 24 h (representative image from 3 independent experiments are shown; similar results were obtained in all 3 independent repeats).

Biocompatibility is a crucial parameter in assessing biomedical materials, requiring minimal or positive effect on cellular viability and the ability to elicit appropriate biological responses in target tissues. Bioactives often demonstrate dose-dependent bioactivity and incompatibility with living cells and tissues. This study employed primary human keratinocytes, fibroblasts, and reconstructed human epidermis (SkinEthic RHE^43,44^) to assess dose-dependent biocompatibility and wound healing potential (Supplementary Figs. 9 and 10). In order to assess improved margin of tolerance with SPB, 2% dose containing 0.8% α-(-)-bisabolol was used. To further validate the hypothesis that the supramolecular structure enhances biocompatibility and to better simulate real-world conditions with topical application, 2% SPB and equivalent concentrations of both the simple mixture and pure α-(-)-bisabolol (0.8% w/w) were topically applied to SkinEthic RHE models, followed by viability evaluation after 18 h of incubation. As illustrated in Fig. 5A, α-(-)-bisabolol treatment at this dosage led to a considerable reduction in model viability (54.9% decrease vs. control, *P* < 0.05). While the direct combination of D-panthenol (0.72%), α-(-)-bisabolol (0.8%), and 0.48% of ethoxydiglycol as a simple mixture, did not change such stress on the epidermal models, the supramolecular format drastically improved, resulting viability, with 85% improvement in tolerance in comparison to α-(-)-bisabolol alone (*P* < 0.05).

Similarly, as shown in Fig. 5C, both α-(-)-bisabolol alone and a mixture of D-panthenol, α-(-)-bisabolol and ethoxydiglycol led to structural insult to the epidermis with equal 0.8% α-(-)-bisabolol dosage, indicated by abnormally organized basal cells, as well as hollow spaces in the upper epidermis. However, the 2% SPB, with the same content of α-(-)-bisabolol, largely maintained the integral *stratum corneum*, as well organized layers of viable keratinocytes. This observation, together with the abovementioned model viability results, suggests higher tolerance and biocompatibility with the SPB format.

With the previous experiment revealing the superior skin penetration properties of SPB, whether it also increases the risk of potential irritation due to improved bioavailability came into question. Between D-panthenol and α-(-)-bisabolol, only the latter was found to be able to induce moderate erythema, despite both being widely used as soothing or anti-inflammatory cosmetic agents^45^. Herein, when the dose of α-(-)-bisabolol was increased to 0.8%, compromised viability and morphological signs of damage were noticed in the RHE models, with or without the presence of D-panthenol. On the other hand, the apparent mildness of SPB was intriguing, which could have been attributed to the variation of physical chemistry property and controlled-release behavior of supramolecule^40^. Collectively, these results demonstrate that SPB’s supramolecular structure confers enhanced biocompatibility while maintaining structural integrity during biological interactions, preventing dissociation into free components. This unique mechanism reduces direct stimulation of cell membrane receptors by individual functional groups. Similar findings were reported by Simar et al.^46^, who observed that ionic liquid/DES-type supramolecular structures exhibit minimal effects on SC proteins, contributing to their low toxicity. Additionally, the fluidizing effect of supramolecular structures on intercellular lipids promotes transcellular transport, thereby minimizing prolonged cellular exposure and direct irritation^47^. Previous studies have also documented the influence of electrostatic potential modulation on toxicity, providing valuable insights for expanding toxicological evaluation parameters^48^.

One of the well-reported benefits of panthenol is its potential to accelerate wound healing and skin repair^14^. Herein, the keratinocyte scratch test was employed to assess the potential of the SPB as a skin repairing agent, and a 1 ng/mL dose of EGF was used as the positive control. As illustrated in Fig. 5B, D, the supramolecular active had a higher repairing effect than the simple mixture and α-(-)-bisabolol alone (*P* < 0.05). This data implies that the keratinocyte re-epithelizing-promoting effect, mainly driven by D-panthenol, was enhanced in the form of SPB, indicating strong potential for therapeutic applications.

### Anti-irritation potential of SPB

**Fig. 6:**
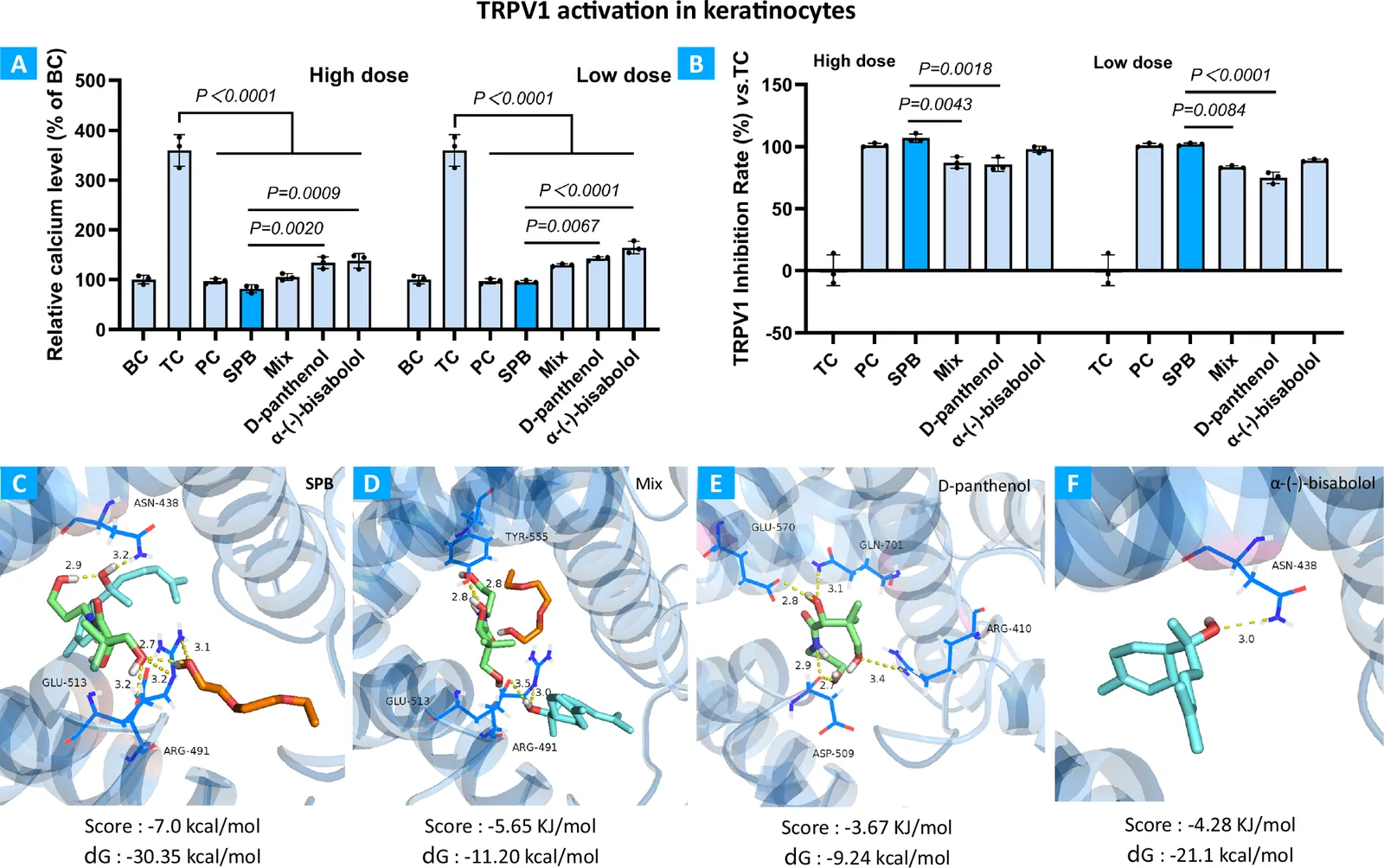
Anti-irritation potential of bioactives in keratinocytes. **A** Intracellular calcium level of keratinocytes triggered with capsaicin and co-treated with the actives. Results are shown as mean ± SD. *N* = 3 biologically independent per group (each experiment used primary keratinocytes isolated from a different donor; the unit of study is the individual isolation). Within each experiment, technical triplicates (three wells per condition) were averaged before statistical analysis. Two-way ANOVA followed by Tukey’s multiple comparison test, two-sided. Groups compared in high dose: SPB *vs*. D-panthenol: Mean difference = −52.30, 95% CI [−89.31, −15.29], q = 6.340, F = 28, adjusted *P* = 0.0020; SPB *vs*. α-(-)-bisabolol: Mean difference = −55.97, 95% CI [−92.97, −18.96], q = 6.784, F = 28, adjusted *P* = 0.0009. Groups compared in low dose: SPB *vs*. D-panthenol: Mean difference = −46.73, 95% CI [−83.74, −9.726], q = 5.665, F = 28, adjusted *P* = 0.0067; SPB *vs*. α-(-)-bisabolol: Mean difference = -69.10, 95% CI [-106.1, −32.09], q = 8.376, F = 28, adjusted *P* = < 0.0001. **B** TRPV1 Inhibition rate (% vs. TC) of bioactives against capsaicin-stimulated calcium influx. Results are shown as mean ± SD. *N* = 3 biologically independent per group, technical triplicates were averaged before statistical analysis. Two-way ANOVA followed by Tukey’s multiple comparison test, two-sided. Groups compared in high dose: SPB *vs*. D-panthenol: Mean difference = 21.33, 95% CI [6.697, 35.97], q = 6.373, F = 24, adjusted *P* = 0.0018; SPB *vs*. Mix: Mean difference = 19.67, 95% CI [5.031, 34.30], q = 5.876, F = 24, adjusted *P* = 0.0043. Groups compared in low dose: SPB *vs*. D-panthenol: Mean difference = 27.00, 95% CI [12.36, 41.64], q = 8.066, F = 24, adjusted *P* = < 0.0001; SPB *vs*. Mix: Mean difference = 18.33, 95% CI [3.697, 32.97], q = 5.477, F = 24, adjusted *P* = < 0.0084. **C**–**F** Molecular docking and binding energies of SPB, Mix, D-panthenol, and α-(-)-bisabolol with TRPV1 (PDB ID:8GF9), respectively.

TRPV1, a nonselective cation channel, serves as a polymodal sensor for various noxious stimuli. Upon activation, TRPV1 mediates calcium (Ca^2+^) influx, which initiates multiple downstream pathological cascades. Primarily, elevated intracellular Ca^2+^ activates phospholipase A2 (PLA2), subsequently promoting prostaglandin synthesis and amplifying inflammatory responses. SPB, as well as individual or mixture of D-panthenol and α-(-)-bisabolol, were applied to keratinocytes with TRPV1 activated, at doses well-under toleration level. The intracellular calcium levels, as well as inhibition rate against capsaicin-triggered control (TC), are captured in Fig. 6A. It was evident that capsaicin, as expected, triggered calcium influx, presumably via activating TRPV1 in keratinocytes, which was ablated by the addition of its known inhibitor trans-4-tert-butylcyclohexanol as a positive control^49^. At doses as low as 4.2 μg/mL and 4.7 μg/mL for D-panthenol and α-(-)-bisabolol, respectively, potent inhibition was also observed individually, as a simple mixture, and in supramolecular form. Specifically, at the low dose, the SPB was able to completely ameliorate calcium influx, showing a superior effect (*P* < 0.05) over a simple mixture of individual components at the equivalent concentrations (Fig. 6B). Detailed dosages are shown in Supplementary Table 5.

With this apparent efficacy, which was superior to a simple mixture of the same D-panthenol and α-(-)-bisabolol at equal doses, it was natural to speculate that the supramolecular format altered the interaction between the bioactive molecules and the intended TRPV1 receptor as a biological target. The docking score^50^ serves as a coarse-grained metric in high-throughput screening for rapid evaluation of potential ligand binding, though with lower precision compared to binding energy calculations. Binding energy refers to the energy released during the interaction between a drug molecule and its target protein^51^, where lower values indicate stronger affinity, greater complex stability, and prolonged residence time. As shown in Fig. 6C–F, molecular docking simulation rendered that the supramolecular structure resulted in lower binding energy at the hypothesized binding site for panthenol, thus suggesting higher affinity. This suggests that SPB may exhibit extended residence time at TRPV1’s active site, effectively blocking access by other ligands or stimuli and thereby enhancing its inhibitory effect. This case study provides novel insights into how supramolecular architectures can potentiate synergistic drug interactions, offering a valuable reference for developing improved inhibitor-class pharmaceuticals.

### Clinical evaluation on anti-erythema efficacy of an emulsion containing SPB

To compare the performance of SPB and a simple mixture in a formulation, a base emulsion containing only 2% of the supramolecular component as the sole active ingredient (Supplementary Table 6) was used in an arm patch test under Sodium Dodecyl Sulfate (SDS) stimulation to evaluate its soothing properties. The viscosities (Brookfield, DV2T: 4#50r, 1924 cp, 18.1%; 1912 cp, 47.8%) and pH (5.9 and 5.7) of the two emulsions (SPB group and simple mixture group) were very similar, with droplet sizes of 93,893 and 36,037 nm (*n* = 3), respectively. This study was approved by the Human Subjects Ethics Committee of Shanxi Biocell General Testing Technology Co., Ltd. (approval No. BXLL2026016). After obtaining informed consent, 31 self-reported sensitive-skinned Chinese volunteers were recruited. The emulsion was applied twice daily for three consecutive days, followed by induction of skin redness with SDS, and then the emulsion was reapplied to record skin recovery. The BC group refers to no application after induction, and untreated means no treatment. Transepidermal water loss (TEWL), erythema index, and images were measured using a TEWL meter (SWL5201, Delfin) and an Antera 3D imager (HC1023Q, Delfin) at T0 (immediately after SDS induction), T1 (immediately after product application), T30 min (30 mins after product application), and T2H (2 h after product application).

**Fig. 7:**
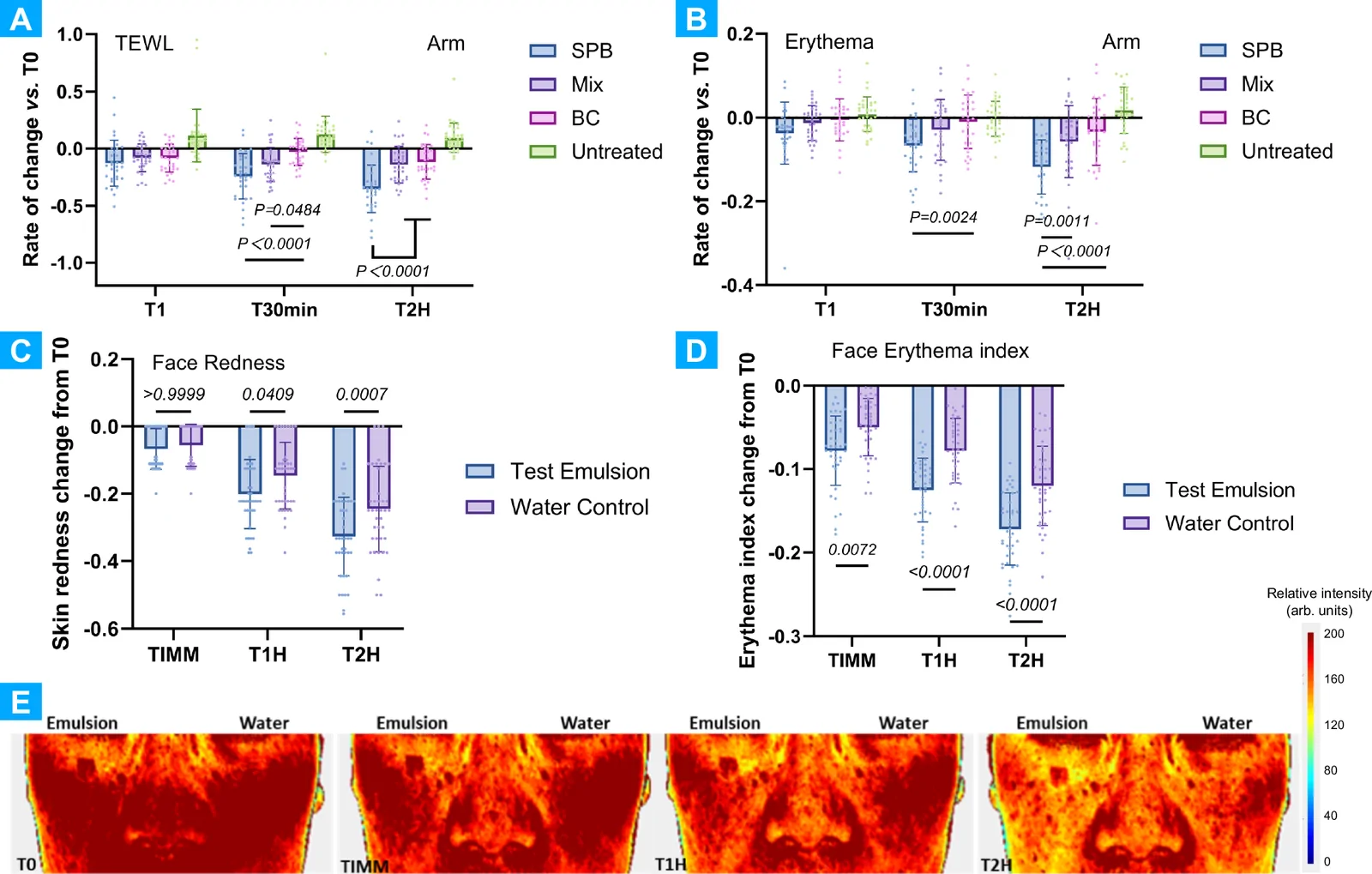
Clinical anti-redness performance of emulsion containing SPB. **A** Arm TEWL changes. Data are presented as mean ± SD. *N* = 31 volunteers (the unit of study is the individual volunteer) and technical triplicates (three measurements per time point) were averaged before statistical analysis. Two-way ANOVA followed by Tukey’s multiple comparison test, two-sided. Groups compared in T30min: SPB *vs*. BC: 95% CI [−0.3230, −0.1048], adjusted *P* < 0.0001; Mix *vs*. BC: 95% CI [−0.2188, −0.0005296], adjusted *P* = 0.0484. Groups compared in T2H: SPB *vs*. BC: 95% CI [−0.3455, −0.1273], adjusted *P* < 0.0001; S*P*B *vs*. Mix: 95% CI [−0.3218, −0.1035], adjusted *P* < 0.0001. **B** Arm erythema index changes. Data are presented as mean ± SD. *N* = 31 volunteers and technical triplicates. Two-way ANOVA followed by Tukey’s multiple comparison test, two-sided. Groups compared in T30min: SPB *vs*. BC: 95% CI [−0.09827, −0.01568], adjusted *P* = 0.0024. Groups compared in T2H: SPB *vs*. BC: 95% CI [−0.1251, −0.04255], adjusted *P* < 0.0001; SPB *vs*. Mix: 95% CI [−0.1017, −0.01907], adjusted *P* < 0.0011. **C** Change from baseline in dermatologist assessment score of skin redness. Data are presented as mean ± SD. *N* = 40 volunteers and technical triplicates. Two-way ANOVA followed by Šídák’s multiple comparison test, two-sided. Groups compared: Test Emulsion *vs*. Water Control. In T1H: 95% CI [−0.1070, −0.001772], adjusted *P* = 0.0403; In T2H: 95% CI [−0.1342, −0.02894], adjusted *P* = 0.0007. **D** Change from baseline in erythema index. Data are presented as mean ± SD. *N* = 40 volunteers and technical triplicates. Two-way ANOVA followed by Šídák’s multiple comparison test, two-sided. Groups compared: Test Emulsion *vs*. Water Control. In TIMM: 95% CI [−0.04999, −0.006067], adjusted *P* = 0.0072; In T1H: 95% CI [−0.06929, −0.02536], adjusted *P* < 0.0001; In T2H: 95% CI [−0.07372, −0.02980], adjusted *P* < 0.0001. **E** Representative images (from 40 volunteers are shown; similar results were obtained in all replicates) taken by Tivi700 of the subject at T0, TIMM, T1H and T2H.

As shown in Fig. 7A and Supplementary Fig. 11A, both SPB and the simple mixture reduced TEWL and repaired the skin barrier, with SPB showing significantly better effects, both immediately and within two hours. Similar results were observed for the erythema index (Fig. 7B and Supplementary Fig. 11B); SPB exhibited a more pronounced anti-redness and repair effect than the simple mixture, with representative images shown in Supplementary Fig. 12. This indicates that preparing supramolecular JANUS eutectics from components with potentially complementary functions in a formulation is a highly promising strategy for benefit enhancement.

To further highlight the efficacy of SPB technology, a facial emulsion containing SPB as the main active substance was formulated and subsequently evaluated in a randomized half-face controlled clinical study for its anti-redness efficacy under infrared light challenge. The study was approved by the Ethics Committee at Shanghai China-norm Quality Technical Service Co., Ltd. (ethical approval No. FSEC2025-15-01). Upon achieving informed consent, 40 Chinese women (mean age of 34.08 yr) with self-declared mild sensitive skin were recruited. They all presented skin redness score ≥ 4 (Griffith 10-point scale) by a dermatologist after infrared light induction. The wavelength of infrared light can penetrate the human dermis to promote blood circulation and induce skin redness. The subject’s face was exposed at a distance of 20 cm from an infrared lamp for 7–8 min at a temperature of 30–40 °C and a wavelength of 760–5000 nm. After induction, the technician applied 0.3 g investigational emulsion on the subject’s half face according to the randomization table and 0.3 g water on the other side. Subjects were subjected to clinical scoring by dermatologists, erythema index measurement by Mexameter® MX 18 and an image taken by TiVi 700 Tissue Viability Imager at the timepoints T0 (immediately after infrared induction), TIMM (10 mins after product application), T1H (1 h after product application) and T2H (2 h after product application).

As demonstrated in Fig. 7C, D, compared with baseline, redness score and erythema index significantly and steadily decreased (*P* < 0.05) after emulsion application with the improvement of 6.72% at TIMM, 20.17% at T1H, and 32.77% at T2H for redness clinical grading, and 7.80%, 12.56% 17.21% reduction in erythema index. More importantly, the emulsion demonstrated superiority over water control treatment, with significantly lower values in both measurements at each timepoint. These results reveal that the emulsion mainly containing SPB could deliver strong anti-redness efficacy in vivo when applied topically in human subjects.

This study establishes a supramolecular Janus Eutectics (supramolecular panthenol bisabolol, SPB) that overcomes the classical penetration‑efficacy‑safety trade‑off in transdermal delivery. Key experimental models—including fresh ex vivo human skin, reconstructed epidermis (SkinEthic RHE), and multiscale computational simulations—collectively demonstrate that SPB: (1) Enhances panthenol delivery (transdermal flux: 1.5‑fold; efficiency: 1.8‑fold) without barrier disruption, (2) Elevates bisabolol biocompatibility (viability ≥95%, 1.7‑fold improvement) and pro‑repair activity, and (3) Exerts integrated anti‑irritation effects, validated in clinical redness reduction and wound closure assays. Mechanistic insights from spectral analysis, molecular dynamics, and docking reveal that the amphiphilic supramolecular structure facilitates skin partitioning and antagonizes TRPV1‑mediated nociception. By unifying hydrophobic and hydrophilic agents into a single biofunctional material, SPB exemplifies a supramolecular strategy to co‑optimize delivery, efficacy, and tolerability—offering a scalable blueprint for next‑generation topical therapeutics.

## Methods

Detailed experimental procedures are described in the Supplementary Information Methods.

### Synthesis of SPB

D-Panthenol and α-(−)-bisabolol (1:1 molar) were heated at 333 K under N_2_ with stirring (300 rpm) for 6 h until homogeneous, then sonicated (300 W, 2 s on/2 s off, ice–water bath, 1 h) with a No. 6 probe. Equimolar ethoxydiglycol was added and the sonication repeated, yielding a pale-yellow, clear supramolecular D-panthenol–α-(-)-bisabolol Janus eutectic (SPB). Simple mixture: the three components (1:1:1 molar) were stirred at room temperature, giving a colorless, clear liquid.

### Characterization

SPB was characterized by ¹H NMR (400 MHz, methanol‑d₄), NOESY (600 MHz), FTIR, and DSC (Tg).

### Simulations

DFT calculations (Gaussian 16, M062X/6-311 g(d)) and IGMH/AIM analyses (Multiwfn 3.8 dev / VMD 1.9.3) were performed. MD simulations (GROMACS) of a ceramide/cholesterol/free fatty acid (1:1:1) bilayer, built with CHARMM-GUI (https://charmm-gui.org/), GROMACS 2020.5, and TIP3P water, were energy-minimized and run in the NPT ensemble (298.15 K, 1 bar) for 10 ns to model transdermal permeation and pulling forces. Docking to TRPV1 (PDB 8GF9) was done with AutoDock 4.2.6, followed by 10 ns MD (GROMACS 2020.5); ligand–receptor interactions were analyzed over the last 10 ns, and visualization was performed using PyMOL 2.6.2.

### Stress test

pH stability: 0.2% SPB or physical mixture solutions were adjusted with glycolic acid/KOH, 48 h at RT. Temperature stability: 0.2% SPB solutions at 4 °C, RT, and 50 °C, sampled on days 0, 5, 10, and 30. Oxidative stability: 0.2% SPB mixed with H_2_O_2_ (95:5 w/w), 48 h. D-panthenol and α-(-)-bisabolol were quantified after each treatment.

### Ex vivo human skin penetration test

#### For ex vivo skin penetration studies

Three skin explants per group were used (*n* = 3). This sample size was chosen based on standard practice for Franz cell experiments in the field, which typically use 3 replicates per condition to account for inter-donor variability^52^.

Franz cells with discarded human abdominal skin (TEWL ≤ 15 g/m^2^/h, receptor fluid PBS 0.1 M, pH 7.4) received ~100 μL sample (~0.108% D-panthenol) non-occluded (0.785 cm^2^, 32 ± 1 °C, 300 rpm). Receptor fluid was sampled at 2, 8 and 24 h with equal volume replacement.

#### Raman

Confocal spectra were acquired from frozen sections with intact dermal–epidermal structures.

#### Quantification

After 24 h, panthenol was extracted from skin surface (PBS rinse), viable tissue (homogenized in PBS), and receptor fluid, then quantified by HPLC; recovery was calculated.

### Mildness test (RHE model)

SkinEthic™ RHE (batch 24ChRHE13) was treated with 32 μL of a 2% solution (equal α-(-)-bisabolol, Supplementary Table 1) and incubated (37 °C, 5% CO_2_, 95% humidity) for 18 h. Viability was assessed by MTT (*n* = 3 independent experiments per group), and one additional tissue was used for histology.

#### For in vitro cell experiments

Three independent biological replicates (*n* = 3 per group) were performed. This sample size is consistent with standard practice in the field and is sufficient to detect the expected large effect sizes based on our preliminary data and previous studies^52^. No formal power analysis was performed for these experiments, as three biological replicates are widely accepted for cell-based assays in this research area.

### Epidermal wound healing potential

Human primary keratinocytes and fibroblasts were cultured in low-calcium medium. Cytotoxicity on keratinocytes was evaluated by MTT assay. For the scratch assay, keratinocytes at 70–80% confluence were treated for 24 h, scratched, and incubated with fresh treatment medium for another 24 h. Wound closure was imaged and quantified; blank (medium only) and positive control (1 ng/mL EGF) were included.

### Anti-irritation potential

Keratinocyte tolerance to serial dilutions was first assessed in 96-well plates by MTT assay. The highest non-toxic dose and a twofold lower dose were then tested for TRPV1 inhibition using an intracellular calcium probe. Based on bisabolol tolerance, 42 μM was used as the high dose and 21 μM as the low dose. Cells pre-loaded with the probe were treated for 1 h. Capsaicin (0.05 μM) served as the trigger and trans-4-tert-butylcyclohexanol (25 μM) as the positive control. Fluorescence intensities were recorded, and relative intracellular calcium levels and inhibition rates were calculated.

### Human volunteer study

A total of 71 healthy volunteers (the first study included 31 participants, 30 females and 1 male, with a mean age of 41.48 years, ranging 22–60; the second study included 40 females with a mean age of 34.08 years, ranging 20–40) were enrolled in this study. Written informed consent was obtained from all volunteers prior to participation. Participants received descriptive compensation, e.g., a $40 gift card for their participation. Sex and gender were not considered as variables in the study design, and no sex- or gender-disaggregated analysis was performed, as the study was not powered for such subgroup comparisons. Transepidermal water loss (TEWL), erythema index, and images were measured using a TEWL meter (SWL5201, Delfin) and an Antera 3D imager (HC1023Q, Delfin). Face erythema index measurement by Mexameter® MX 18 and image taken by TiVi 700 Tissue Viability Imager.

Feasibility considerations and the sample sizes used in similar published studies, which typically include more than 15 subjects, better facilitate the detection of statistical differences, and have reported significant findings with comparable outcome measures^52^. A sample size of 31 or 40 is therefore considered sufficient for the exploratory nature of this study.

### Statistical analyses

Statistical analysis was performed using GraphPad Prism software. For numerical parameters, normality and equal variance were first verified by the Shapiro-Wilk and *F*-test, then subjected to either Analysis of variance (*ANOVA*) or Student’s *t*-test. Post-hoc comparisons were conducted with Dunnett, Tukey or Šídák’s adjustments depending on comparison framing. Statistically significant differences were highlighted by asterisks. For box-and-whisker plots, center line = median, box limits = 25th and 75th percentiles, whiskers = min to max or ±1.5 × IQR, points = individual data points or outliers.

### Reporting summary

Further information on research design is available in the Nature Portfolio Reporting Summary linked to this article.

## Supplementary information

Supplementary Information

Reporting Summary

Transparent Peer Review file

## Data Availability

The raw data generated in this study have been deposited in the Figshare repository at https://doi.org/10.6084/m9.figshare.31082338. Source data is available for Figs. 1–7 and Supplementary Figs. 1–11 in the associated source data file. All data generated or analysed during this study are available within the article and its supplementary information files.
