# Supplementary material for: Supramolecular janus eutectic engineered from panthenol and bisabolol overcomes transdermal delivery paradoxes
Source: Nat Commun. 2026 Sep 8;17:11599. doi: 10.1038/s41467-026-77613-x (PMC13751618; doi:10.1038/s41467-026-77613-x)
Supplement: Supplementary file 1 [file 41467_2026_77613_MOESM1_ESM.pdf]

Supplementary Information

Supramolecular Janus Eutectic Engineered from Panthenol and Bisabolol Overcomes Transdermal Delivery Paradoxes

Hao WANG[1], Qingsheng TAO[2], Xiaofeng HE[2], Kung Chi HSU[2], Yu GAO[2], Delphine KEROB[3], Wei LIU[4], Zhenyuan WANG[5], Nan HUANG[2], Jiaheng ZHANG[1]

[1]Sauvage Laboratory for Smart Materials, School of Materials Science and Engineering, Harbin Institute of Technology (Shenzhen), Shenzhen, China, 518055

[2]L'Oreal Research & Innovation China, Shanghai 201206, China

[3]La Roche-Posay Laboratoire Dermatologique, Levallois Perret 92300, La Roche-Posay, France

[4]Department of Dermatology, The General Hospital of Air Force PLA, Beijing 100142, China

[5]Shenzhen Shinehigh Innovation Co., Ltd, Shenzhen 518055, China.

E-mail: zhangjiaheng@hit.edu.cn, frank.huang@loreal.com, wangzhenyuan@shinehigh.cn

Hao WANG, Qingsheng TAO, and Xiaofeng HE contributed equally to this work.

Methods

1. Synthesis of SPB

The D-Panthenol (purity >98%; Hangzhou Xinfu, Zhejiang, China) and α-(-)-bisabolol (purity >99%; Givaudan) were in a 1:1 molar ratio were heated in a water bath at 333 K on a heated magnetic stirrer (IKA RCT basic) under nitrogen protection (10 L cylinder, high-purity nitrogen, Shenzhen Shenter Industrial Gas Co., Ltd., China) with stirring at 300 rpm for 6 h until no phase separation occurred. The beaker was then placed in an ultrasonic cell crusher (SCIENTZ-IID, Ningbo Xinzhi, China); a No. 6 horn (probe) was immersed to half the depth of the liquid, centered, and ultrasonication was performed at 300 W for 1 h (accompanied by an ice-water bath) with an on/off cycle of 2 s on / 2 s off. Then an equimolar amount of ethoxydiglycol (Gattefossé SAS) was added, and the above process was repeated to obtain a resultant supramolecular D-panthenol-α-(-)-bisabolol Janus eutectic (SPB) as a pale yellow, clear, transparent liquid.

Simple mixture: D-Panthenol, α-(-)-bisabolol, and ethoxydiglycol were mixed at a 1:1:1 molar ratio at room temperature (RT), stirred briefly with a glass rod without any instrumental assistance, yielding a colorless, clear, transparent liquid.

2. General characterization of SPB

^{1}H NMR spectra were recorded on a Bruker Avance Neo 400 MHz spectrometer (Faellanden, Switzerland) using deuterated methanol. NOESY was obtained by an Agilent DD2 600 MHz spectrometer. FTIR spectra were recorded using a Thermo Nicolet 380 spectrometer (Madison, WI, USA). The glass transition temperature (Tg) was determined using a Mettler Toledo DSC3 differential scanning calorimeter (Schwerzenbach, Switzerland).

3. Simulations

The density functional theory (DFT) calculations were performed using Gaussian 09 software at the M062X/6-311g(d) level. The interionic interactions of SPB were analyzed by Multiwfn and VMD softwares based on the independent gradient model based on Hirshfeld partition (IGMH) and atoms-in-molecules (AIM) methods[1-3].

The molecular dynamic simulations were conducted by GROMACS software[4]. The skin barrier was modeled to a bilayer of ceramide (CER), cholesterol (CHOL), and free fatty acid (FFA) with a molar ratio of 1:1:1[5]. CHARMM36m was employed to generate the topology parameters of SPB, CER, CHOL, FFA[6]. The atomistic coordinates of the molecules were added into a cubic box, which was solvated with the tip3p water model to construct D-panthenol/skin complex with pure water using the membrane builder module in CHARMM-GUI[6]. In the energy minimization step, the conjugate gradient algorithm was utilized to remove irrational intramolecular contacts and converge the maximum force to <100 kJ mol^{-1} nm^{-1} . The simulation was conducted in the NPT ensemble, with a pressure of 1.0 bar coupled isotropically with 4.5×10^{-5} compressibility and a coupling constant of 12.0 ps under the Parrinello-Rahman algorithm. The temperature was set to 298.15 K and coupled with a coupling constant of 1.0 ps under the V-rescale algorithm. The short-range interaction cutoff distance was set to 1.0 nm, and the Particle Mesh Ewald algorithm was utilized to compute the long-range interaction. Finally, a 10 ns run of the constrained simulation was executed with a time step of 2 fs for each system to generate the transdermal process of SPB and calculate the pulling forces.

Molecular docking and molecular dynamic simulations were performed[7]. In brief, the molecular docking of SPB, D-Panthenol, α-(-)-bisabolol, and mixture to the TRPV1 receptor (PDB ID: 8GF9) was performed using the AutoDock 4.2.6 program, and MD simulations were performed using GROMACS 5.0.2 program based on the optimized structures. After 10 ns, the root-mean-square deviation stabilized, indicating that the system reached equilibrium. Thus, the interactions between the ligands and TRPV1

were analyzed based on the last 10 ns of the MD simulation trajectory.

4. Stress test

pH stability test: 0.2% SPB or physical mixture aqueous solutions were adjusted to pH gradients using glycolic acid or KOH (Macklin, Shanghai, China). After 48 hr at room temperature (RT), the contents of D-panthenol and α-(-)-bisabolol were determined.

Temperature stability test: 0.2% SPB aqueous solutions were stored at 4°C, RT, and 50°C. The contents of D-panthenol and α-(-)-bisabolol were measured on days 0, 5, 10, and 30.

Oxidant stability test: 0.2% SPB aqueous solution was mixed with hydrogen peroxide at a mass ratio of 95:5. After 48 hr, the contents of D-panthenol and α-(-)-bisabolol were determined.

5. *Ex vivo* human skin penetration test

A Franz cell diffusion system comprised of TK-12D transdermal absorption diffusion instrument and diffusion cells (Kaikai, Shanghai, China) was employed, with surgically discarded human skin (BioCell, Shaanxi, China) positioned in individual diffusion cells and phosphate buffer saline (PBS) being used as receptor fluid. Adult female abdominal skin discards from liposuction procedure were employed for the penetration study. A total of three donors were included, with one replicate per treatment from each donor. Due to privacy considerations, donor age was not disclosed. In order to control the quality of skin explants, trans-epidermal water loss (TEWL) after conditioning in the Franz cell system was measured to ensure integrity of the skin sheets, with an upper limit set at 15 $g/m^2/hr$. For PBS receptor phase was 0.1M with pH of 7.4.

With an effective donor chamber surface area of 0.785 cm^2, a volume of ~100 μL sample, containing ~0.108% of D-panthenol, was applied topically. It was non-occluded after each treatment was applied topically at a volume of 100 μL followed with glass stick smearing. Receptor fluid was sampled at 2, 8, and 24 hrs (2 mL with equal volume

replacement), while an electromagnetic stirrer was maintained at 300 rpm for each cell at a constant temperature of (32 ± 1) °C.

5.1 Raman spectroscopy detection experiment

The confocal Raman spectrometer (inVia reflex, Renishaw, UK) was calibrated by silicon wafer before detection. The quartz gold-plated glass slides with frozen sections were placed on the sample table. Under the microscope, the parts with no separation of the dermis and epidermis, no damage to the skin surface, no folds, and well-defined structures were selected, and their single spectra were measured.

The Raman spectral data set was obtained by scanning the sample with the selected range centered on the point with prominent characteristic peaks and relatively small noise.

5.2 Quantitative analysis experiment

After 24 hr of treatments, panthenol content was extracted from the surface, living skin tissue, and receptor fluid, and thereafter quantified using a high-performance liquid chromatography (HPLC, Agilent, US). In detail, the surface of skin was repeatedly rinsed with 1 mL of PBS, while living skin tissue was removed around the edge of the donor chamber with a sterile scalpel, cut into small pieces, and eventually extracted in 1 mL PBS using a KQ3200E ultrasonic cleaner (40KHz, Kunshan Shumei, CN) at 150W for 60 mins.

The quantity of panthenol in each compartment was calculated by multiplying volume of extracted solution and corresponding concentrations. Recovery of panthenol for each sample was calculated by dividing the summary of quantity of three compartments with pre-weighted loading dose dividing.

6.Mildness test using RHE models

SkinEthic™ RHE is a commercialized reconstructed epidermis model with well differentiated basal layer, spinous layer, granular layer and horny layer. The present test

was carried out on one batch (24ChRHE13) of SkinEthic™ RHE model produced by EpiSkin China.

In order to apply topically, the test actives were diluted in ultrapure water with equal dosage of α-(-)-bisabol, with details laid out in Supplementary Table 1.

Supplementary Table 1. Test materials used for topical treatments in SkinEthics® RHE models.

Active tested	Composition and mass ratio	Test dose
**SPB**	D-panthenol : α-(-)-bisabolol : ethoxydiglycol = 36:40:24	2% (total w/w, 0.8% α-(-)-bisabolol)
**Simple Mixture**	D-panthenol : α-(-)-bisabolol : ethoxydiglycol = 36:40:24	2% (total w/w)
**α-(-)-Bisabolol**	α-(-)-bisabolol : ethoxydiglycol = 40:24	1.28% equiv (0.8% α-(-)-bisabolol w/w)

A volume of 32 μL formula (2%) was topically applied on RHE models, followed by 18 hours of incubation at 37°C, 5% CO2 and 95% humidity. Three replicates of RHE tissue models were harvested and subsequently assessed for viability using standard MTT assay[8]. An additional RHE tissue was used for histological morphology assessment.

7. Epidermal wound healing potential evaluation

Human primary keratinocytes and fibroblasts were maintained in corresponding low-calcium (60 μM) growth medium (all from Biocell Biotechnology, Guangdong, China). The cytotoxicity of D-panthenol, α-(-)-bisabolol, and SPB on human keratinocytes was determined using the MTT assay, as described by a previous study[9].

Keratinocytes cultured in 6-well-plates were treated with medium containing treatments at a volume of 2 mL per well upon reaching 70-80% confluence. Blank control was treated with normal medium while positive control contained 1 ng/mL epidermal growth factor (EGF, Life Technologies, USA). After 24 hr of incubation. The cells were scratched with pipette tips and refreshed for treatment medium. Microscopy images were taken after another 24 hr incubation and the areas recovered from the scratch was calculated

as numerical parameter for comparisons.

8.Anti-irritation potential evaluation

First, 2D keratinocyte tolerance of the bioactives were characterized in 96-well culture plates, with 200 μL of medium containing actives in serial dilutions administered to the cells upon reaching 50-60% confluence. Media was removed after 24 hr of incubation, followed by the addition of MTT working solution (500 μg/mL in fresh medium) and another 4 hr of incubation. Finally, the medium was discarded and cells were dissolved by 150 μL DMSO for OD value measurement at 490 nm.

Then, the actives at the highest non-toxic dose, plus one lower dosages with 2× dilution factors were selected for transient receptor potential V1 (TRPV1) inhibition assay, intended to demonstrate any potential dose-dependency in benefits, using intracellular calcium probe method[10]. As bisabolol was tolerated well at 40 μM but not at 50 μM[11], a dosage of 42 μM (9.4 μg/mL) was used for high dose, and half of that as low dose (4.7 μg/mL). Briefly, the keratinocytes were pre-loaded with the fluorescent probe (need information on the probe from Biocell) through 45 min incubation at 500 μL volume in 24-well plates. Once the probes were preloaded and media was discarded, 500 μL treatments were applied to individual wells with DPBS as blank control (BC), capsaicin (0.05 μM) as triggered control (TC), and trans-4-tert-butylcyclohexanol (25 μM) as positive control (PC) inhibitor. Fluorescence intensity was quantified after 1 hr of reaction, with relative intracellular calcium level (representing activation of TRPV1 channel) and inhibition rate respectively calculated as:

1) Relative intracellular calcium level = $\frac{\text{TC or sample or PC}}{\text{NC}} \times 100\%$

2) Inhibition rate (%) = $\frac{\text{TC} - \text{Sample}}{\text{TC}} \times 100\%$

9. Mexameter® MX18 methodology

Measurement conditions (pressure, site, repetitions): The manufacturer’s instructions for the Mexameter® MX18 do not specify a quantitative pressure value. However, the

spring-loaded probe head is designed to support consistent contact pressure on the skin. To ensure measurement reproducibility in practice, a standardized operating procedure was followed, whereby the probe was positioned perpendicular to the skin surface and applied with a firm, fast, and controlled movement to ensure full contact of the measurement surface with the skin. Triplicated Mexameter measurements was taken on the center of each subject's right or left cheek (at the intersection of lines extending down from the outer corner of the eye and horizontally across the bottom of the nose) on the opposite side of face as Vapometer measurements.

Environmental control (temperature, humidity): According to record from whole clinical period : Temperature: 21.2 ± 0.9°C; Humidity: 51.4 ± 2.3 %

10.Statistical analyses

The Witec data analysis software was used to obtain a clearer spectrum through spectral range selection, removal of cosmic rays, polynomial fitting to remove baselines, and Savitzky-Golay method to smooth the spectrum. For the Raman spectroscopy dataset, univariate analysis was used to extract the compositional information of the experimental samples. The Witec data analysis software completed univariate imaging, that was, Raman spectral imaging. The pseudo-color image reconstruction could be performed by using the comprehensive intensity of the characteristic peak of a substance in the sample scanning spectrum. The intensity of the characteristic peak was related to the brightness of the Raman image color.

Origin software was used to analyze the single-spectral data set, drew the full-layer multi-point Raman scanning spectrum of the skin and reconstruct the Raman pseudo-color map after large-scale scanning, and semi-quantitatively analyzed the characteristic peaks of the substance.

For numerical parameters, normality and equal variance were first verified by Shapiro-Wilk and F-test first, then subjected to either Analysis of variance (ANOVA) or

Student's t-test. Post-hoc comparisons were conducted with Dunnett or Tukey adjustments depending on comparison framing. Statistically significant differences were highlighted by asterisks (* $p<0.05$, ** $p<0.01$, *** $p<0.001$). Such statistical analyses and diagram making was conducted using GraphPad Prism software."

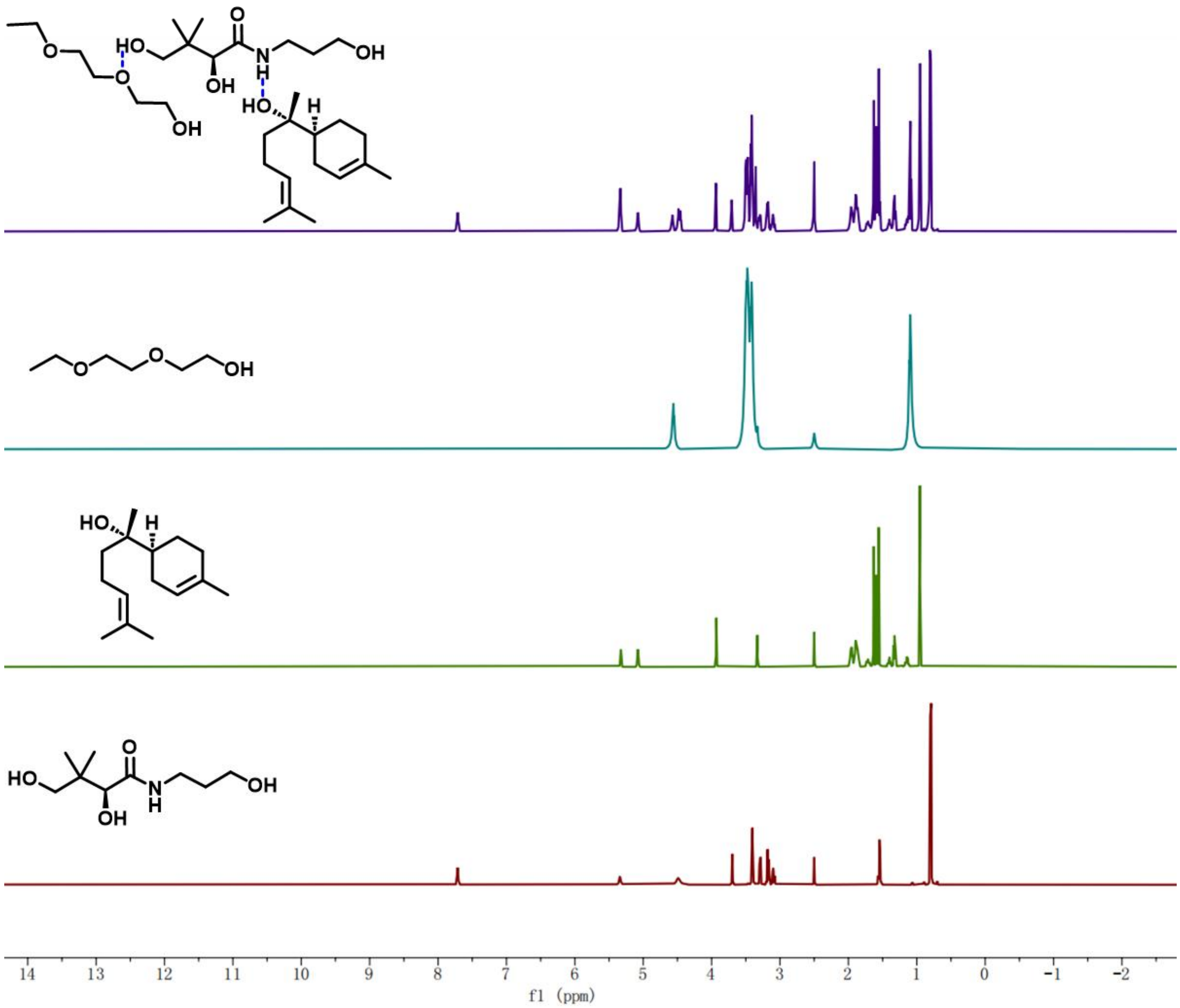

Supplementary Fig. 1 H[1] NMR of SPB components

Supplementary Table 2. Stress test (pH) of SPB (48 hr)

Sample	pH	D-Panthenol content/ppm	D-Panthenol Retention	α-(-)-Bisabolol content/ppm	α-(-)-Bisabolol Retention
0.2% SPB	BC-1	723.70	100%	744.31	100%

aqueous solution	pH=3	705.88	97.54%	734.10	98.63%
	pH=4	716.34	98.98%	740.97	99.55%
	pH=5	719.29	99.39%	741.50	99.62%
	pH=6	718.26	99.25%	738.44	99.21%
	pH=8	712.07	98.39%	737.74	99.12%
	pH=9	696.13	96.19%	743.84	99.94%
	pH=10	660.83	91.31%	741.97	99.69%
	pH=11	558.43	77.16%	746.30	100.27%
0.2% corresponding simple mixed aqueous solution	BC-2	730.86	100.00%	762.74	100.00%
	pH=3	706.04	96.60%	727.54	95.39%
	pH=4	713.49	97.62%	742.03	97.28%
	pH=5	713.83	97.67%	740.90	97.14%
	pH=6	714.21	97.72%	740.93	97.14%
	pH=8	709.37	97.06%	745.18	97.70%
	pH=9	681.74	93.28%	748.29	98.11%
	pH=10	656.60	89.84%	746.11	97.82%
	pH=11	513.89	70.31%	745.07	97.68%

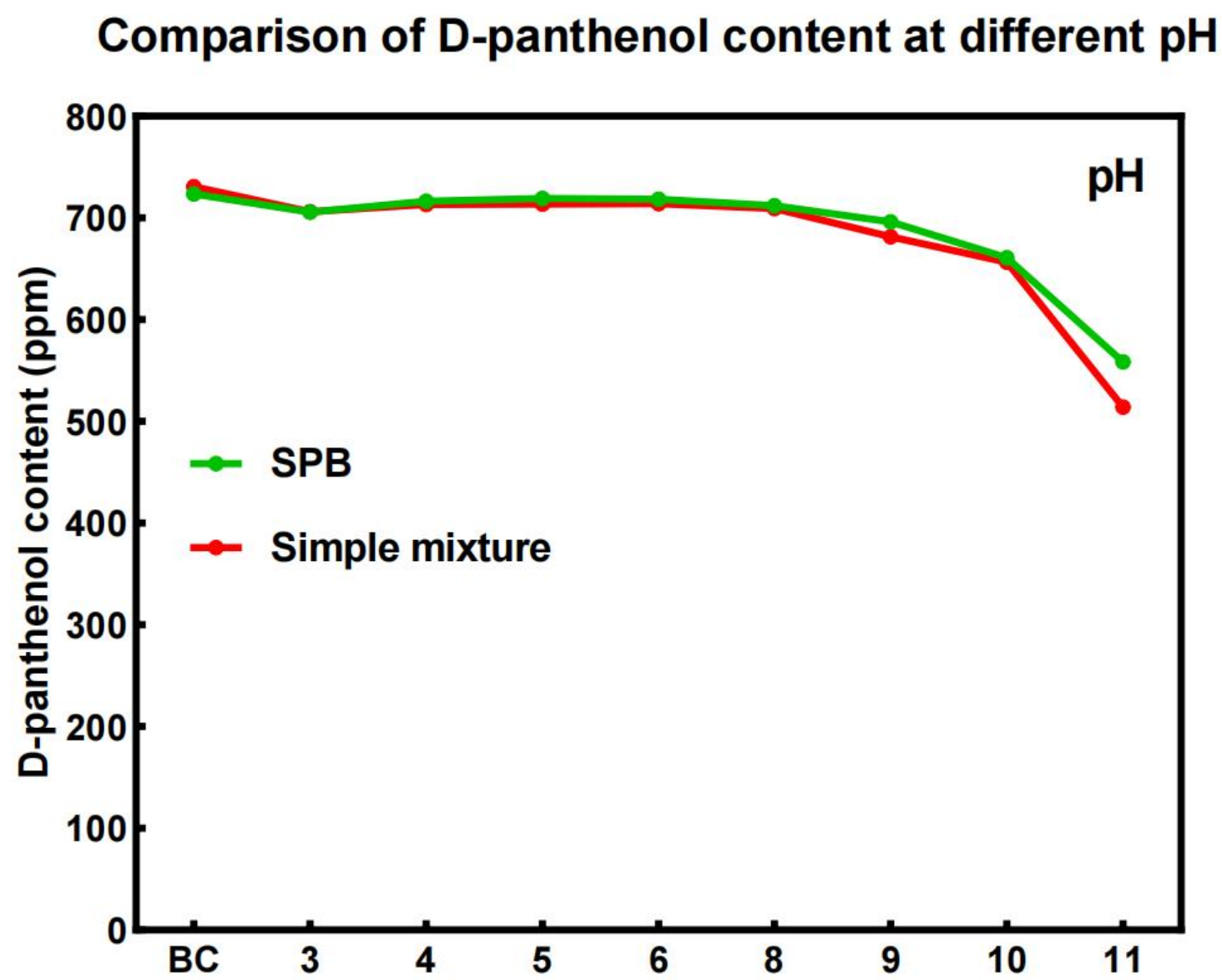

Supplementary Fig. 2 Stress test (pH) of SPB

Supplementary Table 3. Stress test (temperature) of SPB

0.2% SPB aqueous solution	Temperature	D-Panthenol content/ppm	D-Panthenol Retention	α-(-)-Bisabolol content/ppm	α-(-)-Bisabolol Retention
0 D	4℃	716.98	99.79%	742.75	100.24%
	RT	718.49	100.00%	740.97	100.00%
	50℃	720.97	100.35%	742.66	100.23%
5 D	4℃	726.85	99.94%	772.89	100.07%
	RT	727.31	100.00%	772.38	100.00%
	50℃	727.53	100.03%	763.74	98.88%
10 D	4℃	749.36	99.64%	784.3	99.96%
	RT	752.1	100.00%	784.61	100.00%
	50℃	753.45	100.18%	777.97	99.15%
30 D	4℃	748.95	99.08%	793.08	99.89%

	RT	755.94	100.00%	793.95	100.00%
	50℃	747.87	98.93%	783.06	98.63%

Supplementary Table 4. Stress test (H_2O_2) of SPB (48 hr)

Sample	D-Panthenol content/ppm	D-Panthenol Retention	α-(-)-Bisabolol content/ppm	α-(-)-Bisabolol Retention
BC	780.53	100.00%	793.01	100.00%
H_2O_2	781.58	100.13%	804.59	100.13%

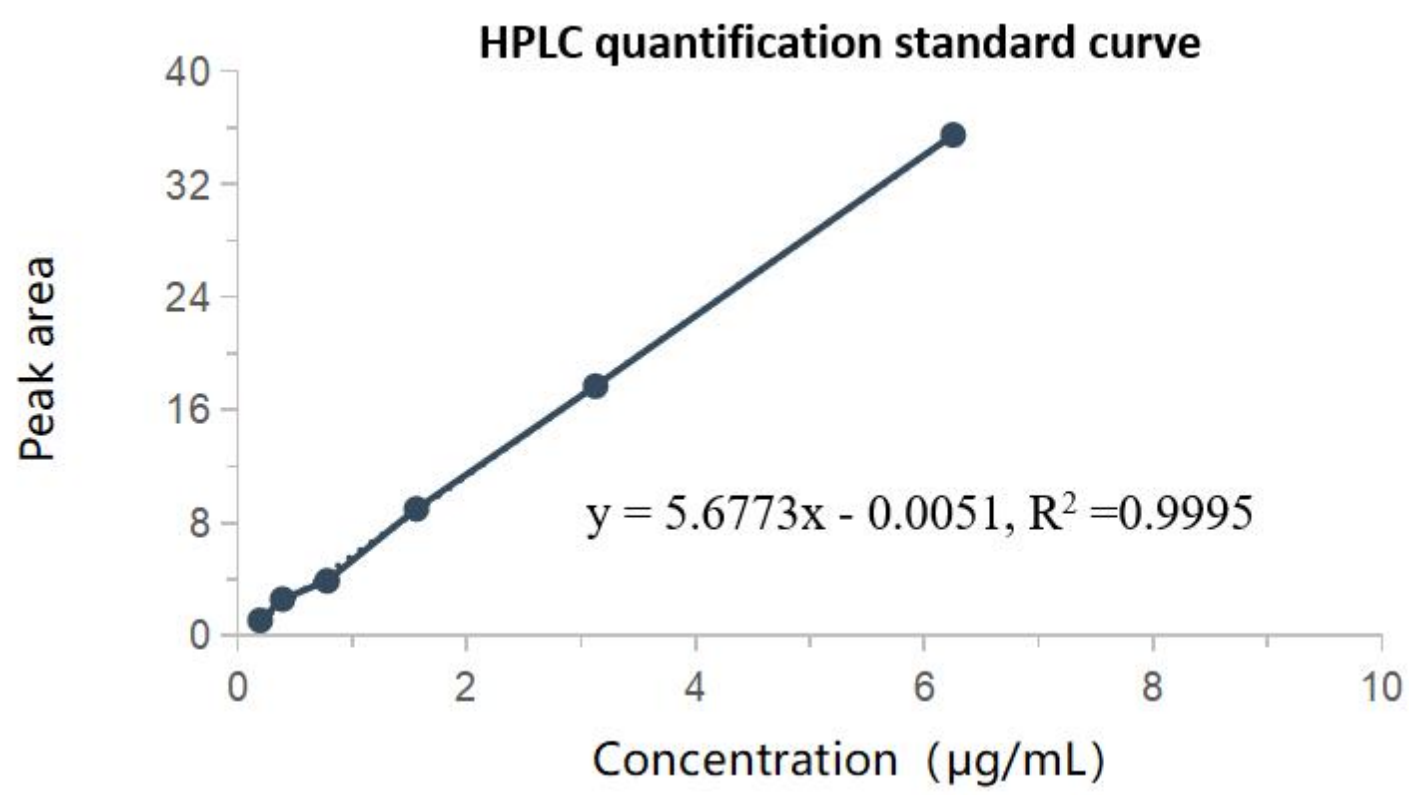

Supplementary Fig. 3 HPLC quantification standard curve of D-panthenol

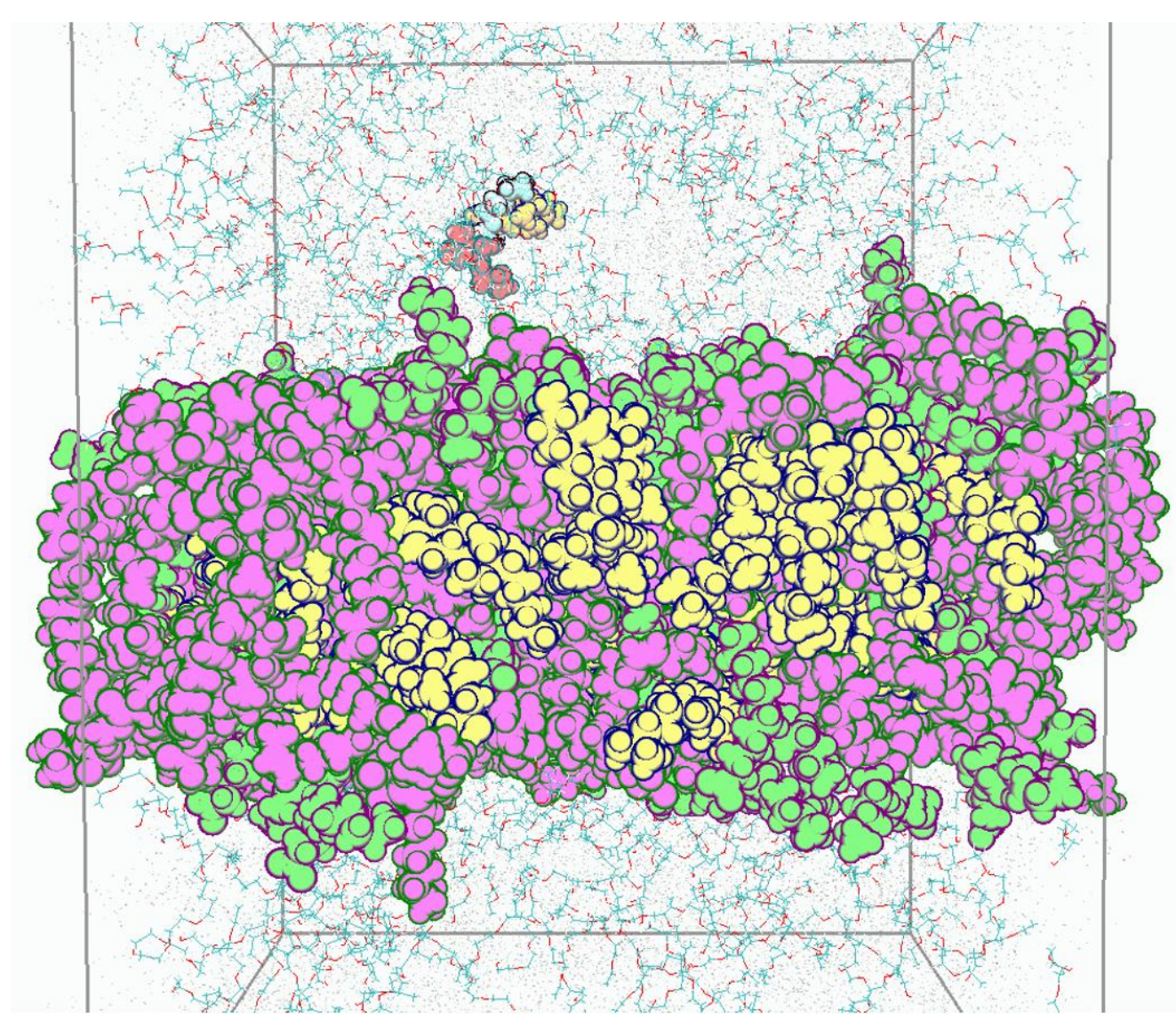

Supplementary Fig. 4 SPB penetration through lipid bilayers process video

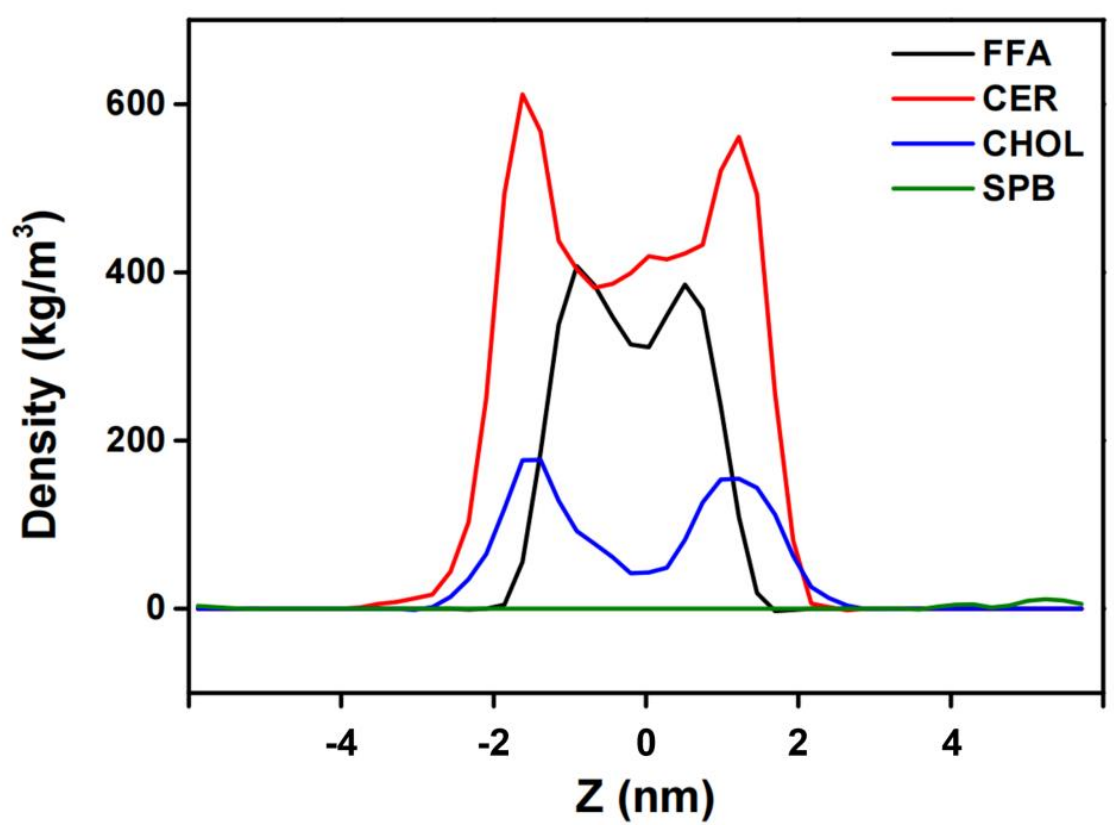

Supplementary Fig. 5 Molecular density distribution of the system during SPB transmembrane permeation. CER: ceramide, CHOL: cholesterol, FFA: free fatty acids, SPB: Supra D-panthenol + α-(-)-bisabolol + ethoxydiglycol.

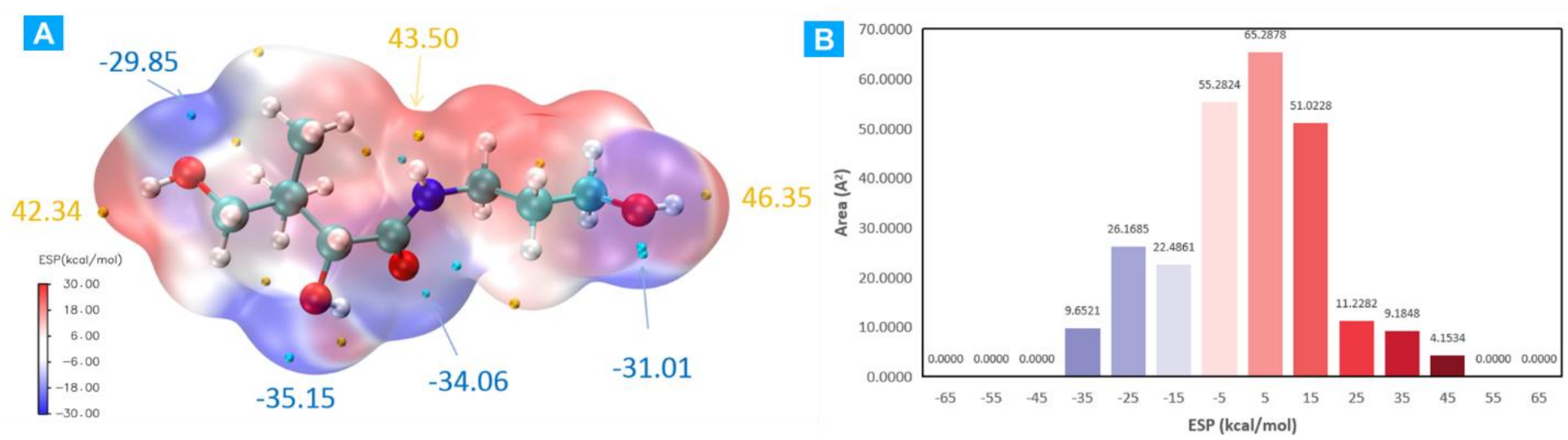

Supplementary Fig. 6 ESP analysis of D-panthenol.

(A) ESP of D-panthenol; (B) Area distribution of ESP for D-panthenol.

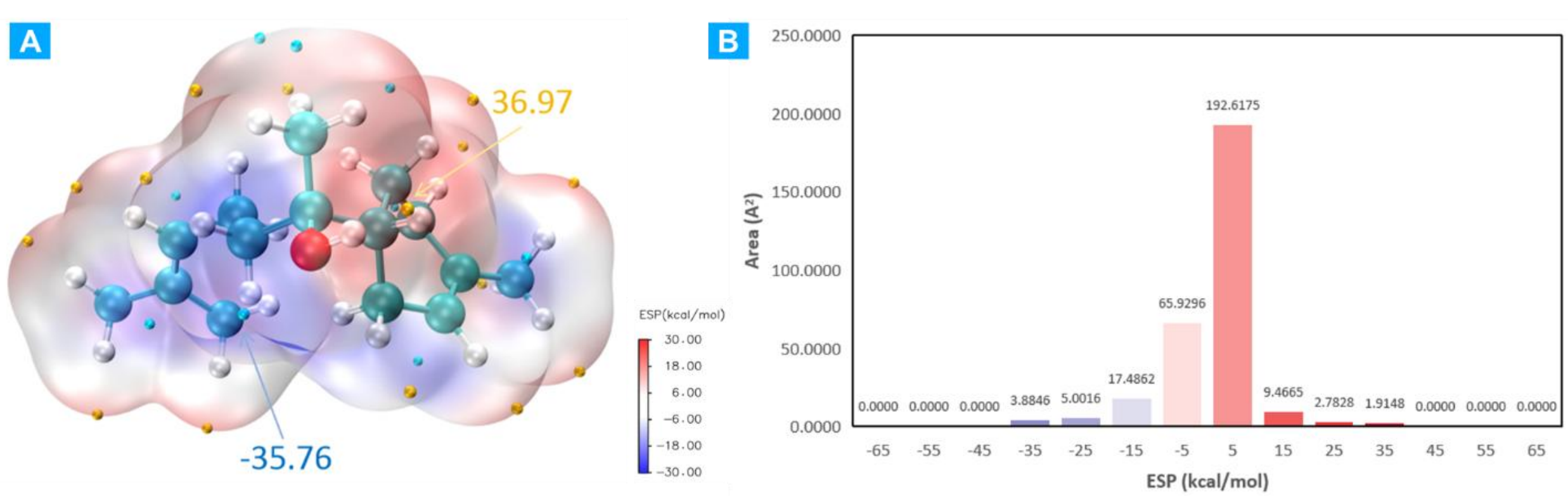

Supplementary Fig. 7 ESP analysis of α-(-)-Bisabolol.

(A) ESP of α-(-)-Bisabolol; (B) Area distribution of ESP for α-(-)-Bisabolol.

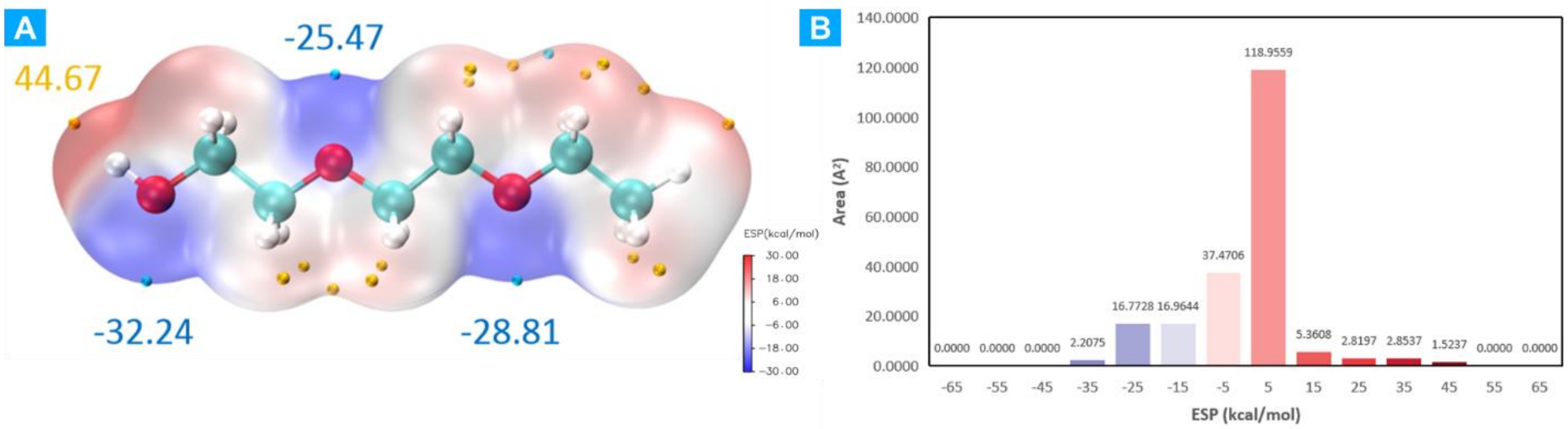

Supplementary Fig. 8 ESP analysis of Ethoxydiglycol

(A) ESP of Ethoxydiglycol; (B) Area distribution of ESP for Ethoxydiglycol.

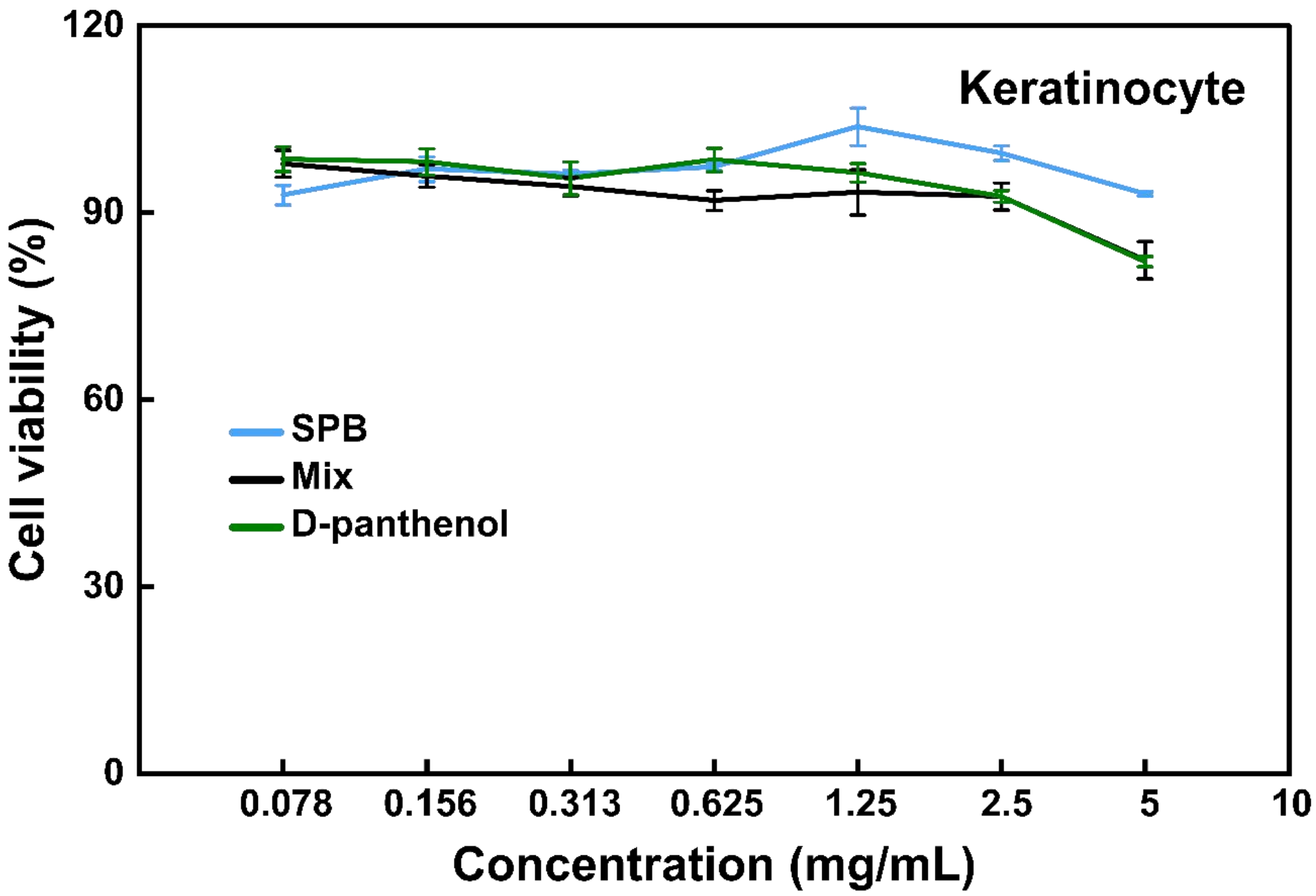

Supplementary Fig. 9 Keratinocytes viability at different concentrations.
Results are shown as mean ± SD. N=3 biologically independent experiments per group.

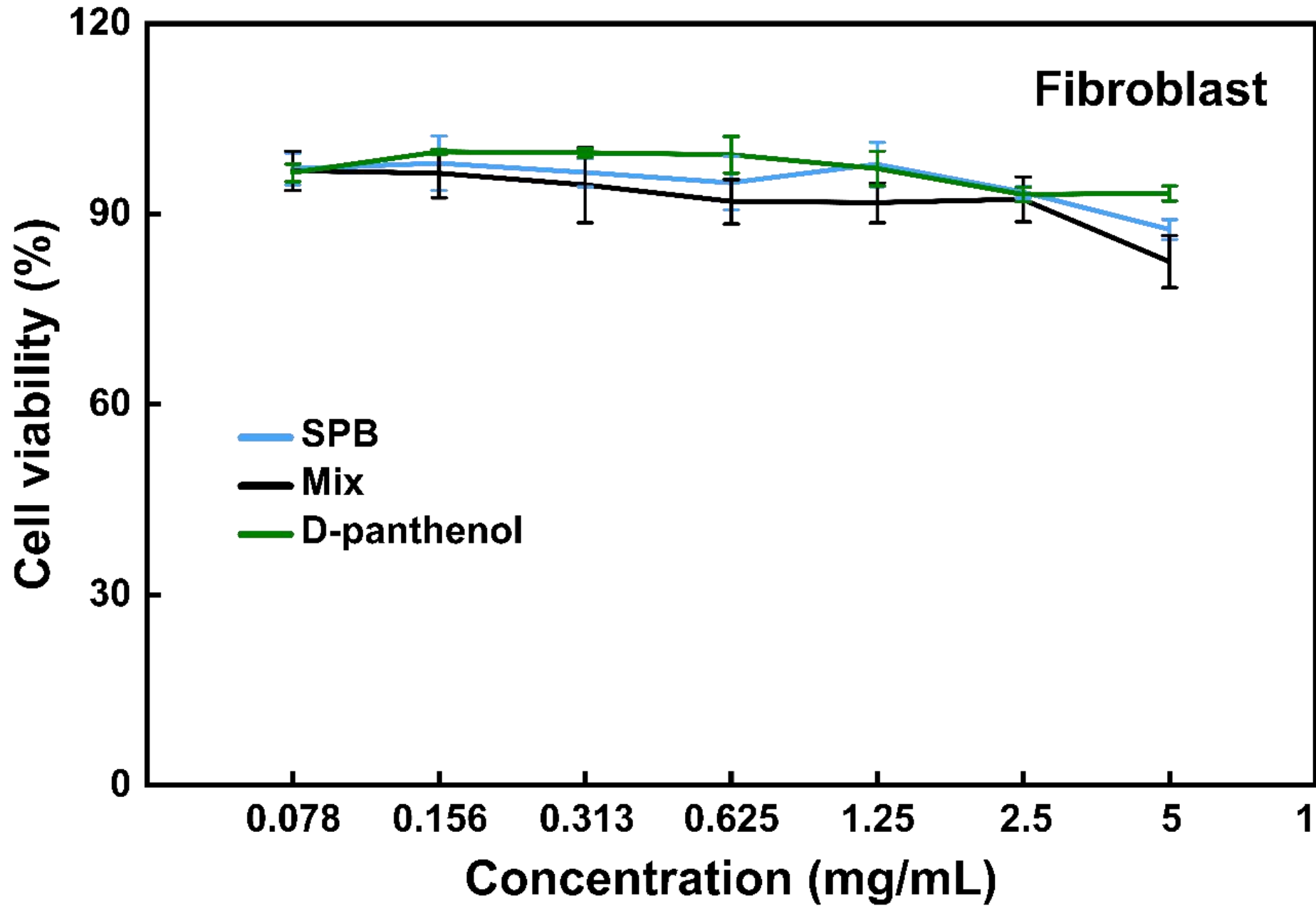

Supplementary Fig. 10 Fibroblasts viability at different concentrations.
Results are shown as mean ± SD. N=3 biologically independent experiments per group.

Supplementary Table 5. Testing doses for TRPV1 inhibition assay in primary human keratinocytes

Groups	Treatments	Dosing concentration	Trigger
Blank control (BC)	/	/	/
Triggered control (TC)	/	/	Capsaicin (0.05 μM)
Positive control (PC)	Trans-4-tert-butylcyclohexanol	25 μM	

	All as μg/mL	***Tota<br>l***	***Pantheno<br>l***	***Bisabolo<br>l***	***Ethoxydiglyco<br>l***
Samples	SPB	23.4	8.4	9.4	5.6
		11.7	4.2	4.7	2.8
	Mixture of D-panthenol +α-(-)-bisabolol	23.4	8.4	9.4	5.6
		11.7	4.2	4.7	2.8
	D-panthenol	14.0	8.4		5.6
		7.0	4.2		2.8
	α-(-)-bisabolol	15.0		9.4	5.6
		7.5		4.7	2.8

Supplementary Table 6. Composition of formula components

**SPB**		**Mix**		*/*
Compents	***Contents (%)***	***Compents***	***Contents (%)***	***Category***
SPB	*2*	*PANTHENOL*	*0.72*	*ACTIVE*
		BISABOLOL	*0.8*	
		Ethoxydiglycol	*0.48*	
SQUALANE	*2*	*SQUALANE*	*2*	*EMOLLIENT*
Caprylic/Capric Triglyceride	*5*	*Caprylic/Capric Triglyceride*	*5*	*EMOLLIENT*
STEARIC ACID	*0.5*	*STEARIC ACID*	*0.5*	*SURFACTANT*

GLYCERYL STEARATE SE	*0.8*	*GLYCERYL STEARATE SE*	*0.8*	*SURFACTANT*
XANTHAN GUM	*0.1*	*XANTHAN GUM*	*0.1*	*POLYMER*
AMMONIUM POLYACRYLOYLDIMETHYL TAURATEPHENOXYETHANOL	*0.4*	*AMMONIUM POLYACRYLOYLDIMETHYL TAURATEPHENOXYETHANOL*	*0.4*	*POLYMER*
PHENOXYETHANOL	*0.2*	*PHENOXYETHANOL*	*0.2*	*Preservative*
GLYCERIN	*3*	*GLYCERIN*	*3*	*SOLVENT*
PENTYLENE GLYCOL	*1.5*	*PENTYLENE GLYCOL*	*1.5*	*SOLVENT*
CITRIC ACID	*QS*	*CITRIC ACID*	*QS*	*pH adjustment*
WATER	*QS*	*WATER*	*QS*	*SOLVENT*

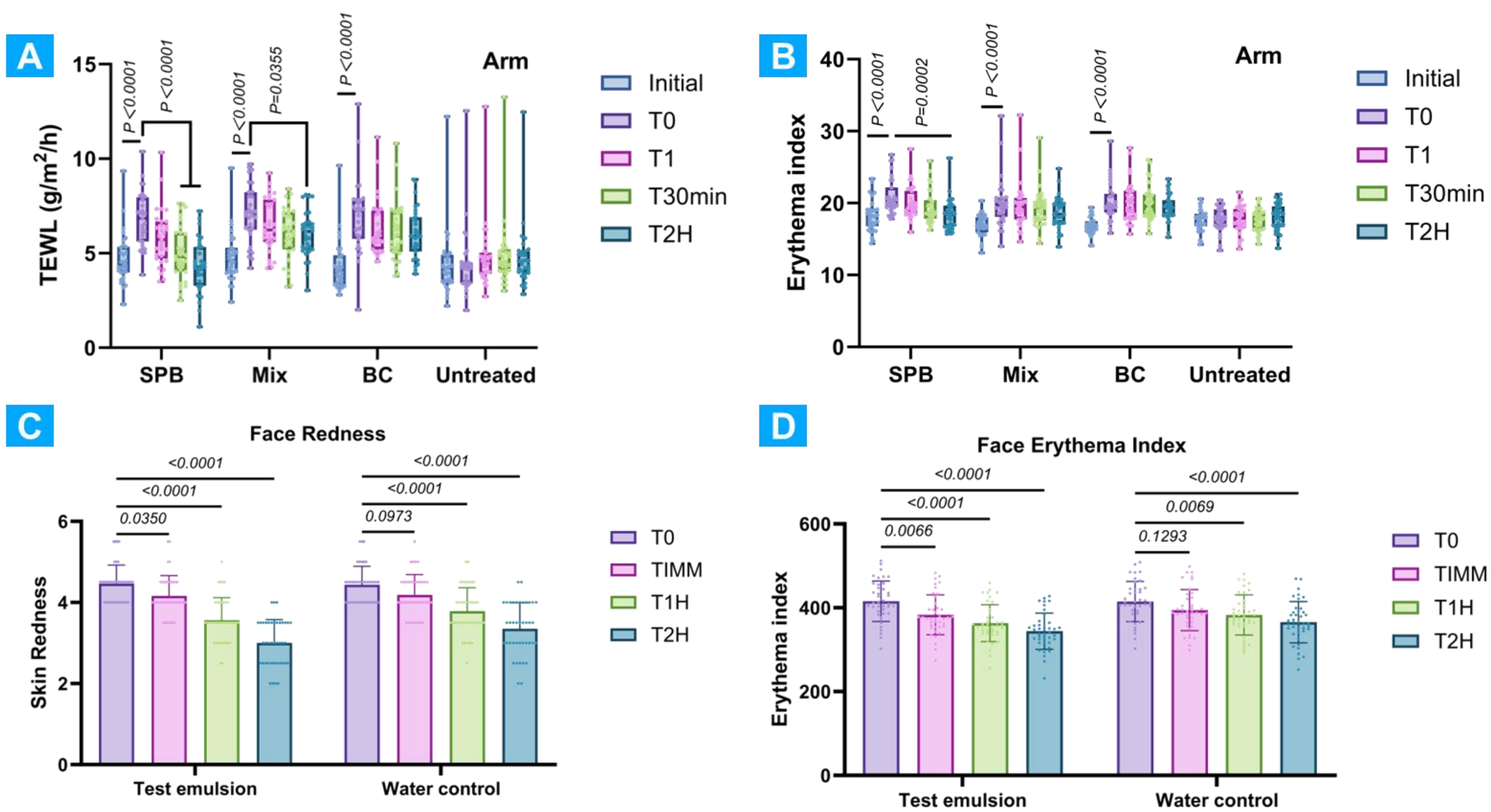

Supplementary Fig. 11 Clinical anti-redness performance of emulsion containing SPB. (A) Arm TEWL changes. Data are presented as mean ± SD. N = 31 volunteers and technical triplicates Two-way ANOVA followed by Tukey's multiple comparison test, two-sided. (B) Arm erythema index changes. Data are presented as mean ± SD. N = 31 volunteers and technical triplicates. Two-way ANOVA followed by Tukey's multiple comparison test, two-sided. (C) Face TEWL changes. Data are presented as mean ± SD. N = 40 volunteers and technical triplicates Two-way ANOVA followed by Dunnett's multiple comparison test, two-sided. (D) Face erythema index

changes. Data are presented as mean ± SD. N = 40 volunteers and technical triplicates.

Two-way ANOVA followed by Dunnett's multiple comparison test, two-sided.

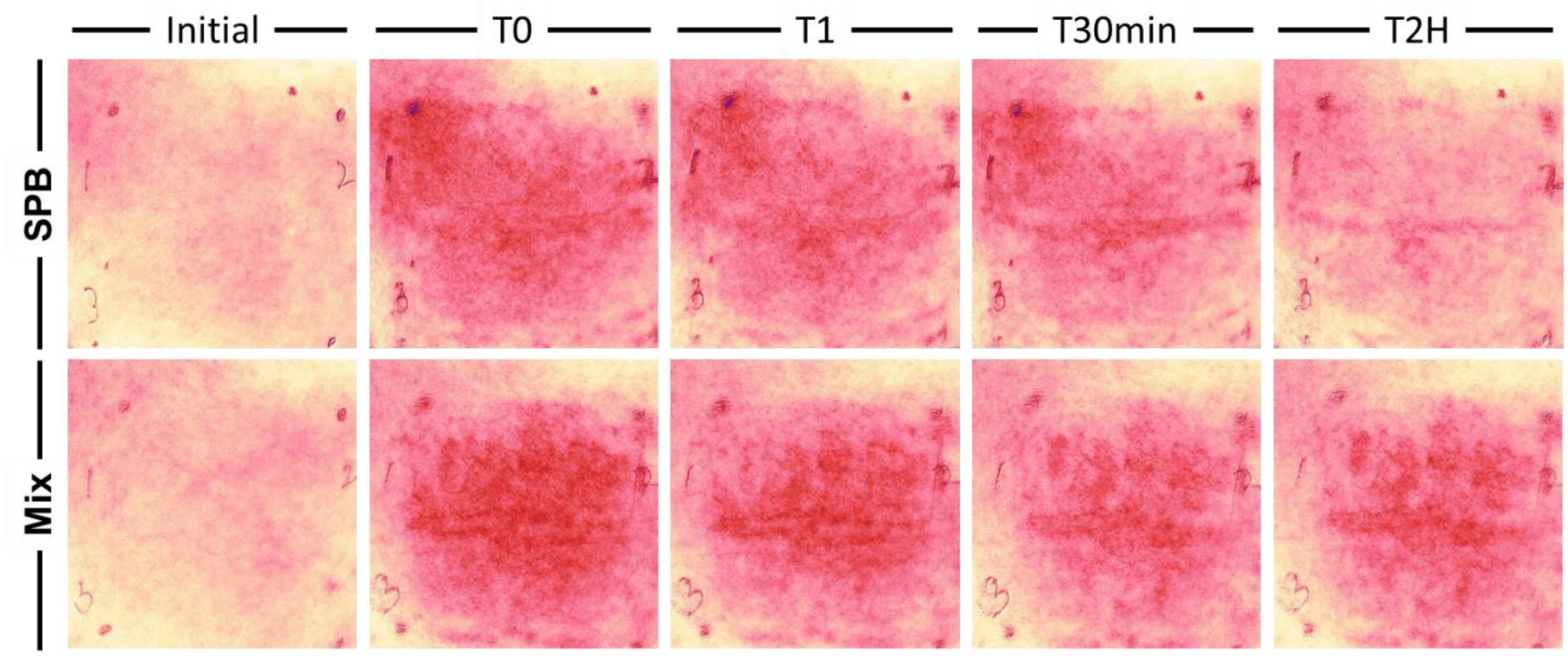

Supplementary Fig. 12 Soothing comparison test between SPB and the simple mixture group (representative images from 31 volunteers are shown; similar results were obtained in all replicates).

Supplementary References

1. Lu, T. & Chen, F. Multiwfn: a multifunctional wavefunction analyzer. *J Comput Chem* **33**, 580-592 (2012).

2. Humphrey, W., Dalke, A. & Schulten, K. VMD: visual molecular dynamics. *J Mol Graph* **14**, 33-38, 27-38 (1996).

3. Lu, T. & Chen, Q. Independent gradient model based on Hirshfeld partition: A new method for visual study of interactions in chemical systems. *J Comput Chem* **43**, 539-555 (2022).

4. Van Der Spoel, D. et al. GROMACS: fast, flexible, and free. *J Comput Chem* **26**, 1701-1718 (2005).

5. Gupta, R., Sridhar, D.B. & Rai, B. Molecular Dynamics Simulation Study of Permeation of Molecules through Skin Lipid Bilayer. *J Phys Chem B* **120**, 8987-8996 (2016).

6. Huang, J. et al. CHARMM36m: an improved force field for folded and intrinsically disordered proteins. *Nat Methods* **14**, 71-73 (2017).
7. Yufei, L. et al. Exploring the effect of surfactants on the interaction between laccase and bisphenol A by molecular docking, molecular dynamics, and energy calculations. *Journal of Molecular Liquids* **382** (2023).
8. Bataillon, M. et al. Characterization of a New Reconstructed Full Thickness Skin Model, T-Skin™, and its Application for Investigations of Anti-Aging Compounds. *Int J Mol Sci* **20** (2019).
9. Wang, M. et al. Sustainable Bioactive Salts Fully Composed of Natural Products for Enhanced Pharmaceutical Applicability. *ACS Sustainable Chemistry & Engineering* **10**, 10369-10382 (2022).
10. Ticona, L.A., Sánchez Á, R., Estrada, C.T. & Palomino, O.M. Identification of TRPV1 Ion Channels Agonists of Tropaeolum tuberosum in Human Skin Keratinocytes. *Planta Med* **87**, 383-394 (2021).
11. Tan, C. et al. The combination of allantoin, bisabolol, D-panthenol and dipotassium glycyrrhizinate mitigates UVB-induced PGE2 synthesis by keratinocytes. *International Journal of Cosmetic Science* **46**, 691-701 (2024).
