# Supplementary material for: Supramolecular janus eutectic engineered from panthenol and bisabolol overcomes transdermal delivery paradoxes
Source: Nat Commun. 2026 Sep 8;17:11599. doi: 10.1038/s41467-026-77613-x (PMC13751618; doi:10.1038/s41467-026-77613-x)
Supplement: Supplementary file 2 [file 41467_2026_77613_MOESM2_ESM.pdf]

nature portfolio

Corresponding author(s): Jiaheng ZHANG

Last updated by author(s): Aug 10, 2026

Reporting Summary

Nature Portfolio wishes to improve the reproducibility of the work that we publish. This form provides structure for consistency and transparency in reporting. For further information on Nature Portfolio policies, see our Editorial Policies and the Editorial Policy Checklist.

Statistics

For all statistical analyses, confirm that the following items are present in the figure legend, table legend, main text, or Methods section.

n/a	Confirmed	
☐	☒	The exact sample size (n) for each experimental group/condition, given as a discrete number and unit of measurement
☐	☒	A statement on whether measurements were taken from distinct samples or whether the same sample was measured repeatedly
☐	☒	The statistical test(s) used AND whether they are one- or two-sided<br>*Only common tests should be described solely by name; describe more complex techniques in the Methods section.*
☒	☐	A description of all covariates tested
☐	☒	A description of any assumptions or corrections, such as tests of normality and adjustment for multiple comparisons
☐	☒	A full description of the statistical parameters including central tendency (e.g. means) or other basic estimates (e.g. regression coefficient) AND variation (e.g. standard deviation) or associated estimates of uncertainty (e.g. confidence intervals)
☐	☒	For null hypothesis testing, the test statistic (e.g. F, t, r) with confidence intervals, effect sizes, degrees of freedom and P value noted<br>*Give P values as exact values whenever suitable.*
☒	☐	For Bayesian analysis, information on the choice of priors and Markov chain Monte Carlo settings
☒	☐	For hierarchical and complex designs, identification of the appropriate level for tests and full reporting of outcomes
☒	☐	Estimates of effect sizes (e.g. Cohen's d, Pearson's r), indicating how they were calculated

Our web collection on statistics for biologists contains articles on many of the points above.

Software and code

Policy information about availability of computer code

Data collection	Transepidermal water loss (TEWL), erythema index, and images were measured using a TEWL meter (SWL5201, Delfin) and an Antera 3D imager (HC1023Q, Delfin). Face erythema index measurement by Mexameter® MX 18 and image taken by TiVi 700 Tissue Viability Imager. The DFT calculations data (e.g., ESP, RDG, IGMH) were obtained using Gaussian 16, Multiwfn 3.8 dev, and VMD 1.9.3. The MD simulation data for the molecular permeation process were obtained from CHARMM-GUI (https://charmm-gui.org/) and GROMACS 2020.5. Docking to TRPV1 (PDB8GF9) was done with AutoDock 4.2.6, and visualization was performed using PyMOL 2.6.2.
Data analysis	Statistical analyses were conducted using Microsoft Excel 2024 (LTSC) (Microsoft Corp., Redmond, WA, USA), WITec Suite FIVE (WITec GmbH, Ulm, Germany), GraphPad Prism 10.1.2 (GraphPad Software, San Diego, CA, USA) and Origin 2025(OriginLab Corporation, Northampton, MA, USA). No custom code was written.

For manuscripts utilizing custom algorithms or software that are central to the research but not yet described in published literature, software must be made available to editors and reviewers. We strongly encourage code deposition in a community repository (e.g. GitHub). See the Nature Portfolio guidelines for submitting code & software for further information.

Data

Policy information about availability of data

All manuscripts must include a data availability statement. This statement should provide the following information, where applicable:

- Accession codes, unique identifiers, or web links for publicly available datasets
- A description of any restrictions on data availability
- For clinical datasets or third party data, please ensure that the statement adheres to our policy

The raw data generated in this study have been deposited in the Figshare repository at https://doi.org/10.6084/m9.figshare.31082338. Source data is available for Figures 1-7 and Supplementary Figures 1-11 in the associated source data file. All data generated or analysed during this study are available within the article and its supplementary information files.

Research involving human participants, their data, or biological material

Policy information about studies with human participants or human data. See also policy information about sex, gender (identity/presentation), and sexual orientation and race, ethnicity and racism.

Reporting on sex and gender	Sex was assigned based on self-report (male/female). No analyses were conducted to compare sexes as the study focused on an all-female population. Gender information was disclosed with the consent of all participants.
Reporting on race, ethnicity, or other socially relevant groupings	Race and ethnicity data were not collected because the study was conducted in a single, ethnically homogenous region and these variables were not relevant to the research question.
Population characteristics	Chinese women aged from 20-60 years old.
Recruitment	Recruitment was conducted through social media. Main Criteria: Chinese women aged from 20-60 years old. Self-declared mild sensitive skin. Self-declared with skin concerns of redness and easy to flushing. Willing to be exposed to infrared light on the facial skin to induce facial redness (can tolerate heat sensation on the face). Subjects received an honorarium of approximately USD 40 upon completion, which helped ensure consistency in the skin characteristics of the participant group.
Ethics oversight	Ethics Committee at Shanghai China-norm Quality Technical Service Co., Ltd.

Note that full information on the approval of the study protocol must also be provided in the manuscript.

Field-specific reporting

Please select the one below that is the best fit for your research. If you are not sure, read the appropriate sections before making your selection.

☒ Life sciences ☐ Behavioural & social sciences ☐ Ecological, evolutionary & environmental sciences

For a reference copy of the document with all sections, see nature.com/documents/nr-reporting-summary-flat.pdf

Life sciences study design

All studies must disclose on these points even when the disclosure is negative.

Sample size	For ex vivo skin penetration studies: Three skin explants per group were used (n = 3). This sample size was chosen based on standard practice for Franz cell experiments in the field, which typically use 3 replicates per condition to account for inter-donor variability.<br>For in vitro cell experiments: Three independent biological replicates (n = 3 per group) were performed. This sample size is consistent with standard practice in the field and is sufficient to detect the expected large effect sizes based on our preliminary data and previous studies. No formal power analysis was performed for these experiments, as three biological replicates are widely accepted for cell-based assays in this research area.<br>For the human volunteer study: A total of 40 volunteers were enrolled. Feasibility considerations and the sample sizes used in similar published studies, which typically more than 15 subjects are included, which better facilitates the detection of statistical differences, and have reported significant findings with comparable outcome measures. A sample size of 31 or 40 is therefore considered sufficient for the exploratory nature of this study.
Data exclusions	No data were excluded from the analysis. All collected data points are reported.
Replication	Each experiment was performed in triplicate (technical replicates) and repeated three times (biological replicates), yielding similar outcomes.
Randomization	For recruitment, all participants received the same questionnaire. The laboratory experiments used a randomized block design with allocation via random number table. The facial test was also a randomized split-face study.
Blinding	The dermatologist performing the procedure, the person scoring the images, and the statistician were blinded to group allocation. Blinding was achieved by coding the samples with random numbers that were revealed only after the final analysis.

Reporting for specific materials, systems and methods

We require information from authors about some types of materials, experimental systems and methods used in many studies. Here, indicate whether each material, system or method listed is relevant to your study. If you are not sure if a list item applies to your research, read the appropriate section before selecting a response.

Materials & experimental systems

n/a	Involved in the study	
☒	☐	Antibodies
☐	☒	Eukaryotic cell lines
☒	☐	Palaeontology and archaeology
☒	☐	Animals and other organisms
☐	☒	Clinical data
☒	☐	Dual use research of concern
☒	☐	Plants

Methods

n/a	Involved in the study	
☒	☐	ChIP-seq
☒	☐	Flow cytometry
☒	☐	MRI-based neuroimaging

Eukaryotic cell lines

Policy information about cell lines and Sex and Gender in Research

Cell line source(s)	Primary human epidermal keratinocytes were isolated from episkin and foreskins of boys aged 6–12, following written informed consent from the donors' parents.
Authentication	The identity and purity of the isolated primary cells were confirmed using cell-type-specific markers: keratinocytes were characterized by positive staining for cytokeratin 14 (K14), and fibroblasts by positive staining for vimentin, with purity exceeding 95% as determined by flow cytometry.
Mycoplasma contamination	All primary cell cultures were routinely tested for mycoplasma contamination using a PCR-based method (e.g., the MycoAlert™ Mycoplasma Detection Kit) and were confirmed to be negative. Cultures were maintained in antibiotic-free medium to facilitate detection.
Commonly misidentified lines (See ICLAC register)	As this study exclusively employed primary human cells derived directly from fresh tissue, and not immortalized or established cell lines, consultation of the ICLAC Register of Misidentified Cell Lines was not applicable.

Clinical data

Policy information about clinical studies

All manuscripts should comply with the ICMJE guidelines for publication of clinical research and a completed CONSORT checklist must be included with all submissions.

Clinical trial registration	FSEC2025-15-01; BXLL2026016.
Study protocol	The study protocols were approved by the Institutional Review Board of Ethics Committee at Shanghai China-norm Quality Technical Service Co., Ltd. and Human Subjects Ethics Committee of Shaanxi Biocell General Testing Technology Co., Ltd. A detailed protocol, see the Supporting Information.
Data collection	Data were collected at the Shanghai China-norm Quality Technical Service Co., Ltd.(Shanghai, CN) between Mar.09, 2025 and Mar.14, 2025. Data were collected at the Shaanxi Biocell General Testing Technology Co., Ltd.(Shanxi, CN) between Mar.20, 2026 and JuN.01, 2026. Recruitment was conducted through social media.
Outcomes	The primary outcomes (erythema characteristics) were: (i) clinical erythema score (Griffith 10-points scale, by blinded dermatologists), (ii) erythema index (Mexameter® MX18), and (iii) tissue viability images (TiVi-700). The secondary outcome was transepidermal water loss (TEWL, SWL5201). All outcomes were measured at T0 (baseline), TIMM (10 min post-product), T1H (1 h), and T2H (2 h). Transepidermal water loss (TEWL), erythema index, and images were measured using a TEWL meter (SWL5201, Delfin) and an Antera 3D imager (HC1023Q, Delfin) at T0 (immediately after SDS induction), T1 (immediately after product application), T30□min (30 mins after product application), and T2H (2 hours after product application).

Plants

Seed stocks	*Report on the source of all seed stocks or other plant material used. If applicable, state the seed stock centre and catalogue number. If plant specimens were collected from the field, describe the collection location, date and sampling procedures.*
Novel plant genotypes	*Describe the methods by which all novel plant genotypes were produced. This includes those generated by transgenic approaches, gene editing, chemical/radiation-based mutagenesis and hybridization. For transgenic lines, describe the transformation method, the number of independent lines analyzed and the generation upon which experiments were performed. For gene-edited lines, describe the editor used, the endogenous sequence targeted for editing, the targeting guide RNA sequence (if applicable) and how the editor was applied.*
Authentication	*Describe any authentication procedures for each seed stock used or novel genotype generated. Describe any experiments used to assess the effect of a mutation and, where applicable, how potential secondary effects (e.g. second site T-DNA insertions, mosiacism, off-target gene editing) were examined.*
