# Supplementary material for: Supramolecular janus eutectic engineered from panthenol and bisabolol overcomes transdermal delivery paradoxes
Source: Nat Commun. 2026 Sep 8;17:11599. doi: 10.1038/s41467-026-77613-x (PMC13751618; doi:10.1038/s41467-026-77613-x)
Supplement: Supplementary file 3 [file 41467_2026_77613_MOESM3_ESM.pdf]

nature portfolio

Peer Review File

Supramolecular Janus Eutectic Engineered from Panthenol and Bisabolol Overcomes Transdermal Delivery Paradoxes

Corresponding Author: Professor Jiaheng Zhang

This file contains all reviewer reports in order by version, followed by all author rebuttals in order by version.

Version 0:

Reviewer comments:

Reviewer #1

(Remarks to the Author)
This study provides a detailed analysis of the advantages and underlying mechanisms of the SPB through a combination of various experimental models and corresponding theoretical calculations. The experimental design is relatively rigorous and closely aligned with practical application scenarios, demonstrating considerable academic merit and promising translational potential. However, several issues remain that require further refinement and clarification.

1. The introduction mentions drawbacks of traditional delivery systems, such as the complexity of preparation, toxicity, and stability issues of nanocarriers. Relevant reports should be added to provide theoretical support.
2. The term “Janus Eutectics” is used in the text; its definition or distinction from DES should be clarified. Additionally, it is recommended to modify expressions like “breakthrough” and “revolutionary” to more reasonable alternatives.
3. The source of logP data and its physical meaning should be explained to ensure logical rigor.
4. The rationale for selecting a 1:1:1 molar ratio for the SPB should be provided. It should also be clarified whether other ratios were attempted and how this specific ratio was optimized.
5. The IGMH analysis needs further elaboration to ensure consistency with hydrogen bond sites and characterization data. Supporting evidence for using ESP distribution to predict amphiphilicity should be supplemented.
6. The experimental design of the stress test should be detailed, including the specific amount used in the test.
7. Figure issues: The numbers in Figure 1C and Figure 2B are not clear enough.
8. The scientific validity and accuracy of the SkinEthic™ RHE model used in the study should be supported with references.
9. The rationale for selecting a 2% concentration in the RHE model tests should be provided.
10. The basis for choosing the two concentrations in the TRPV1 inhibition rate experiment should be explained.
11. Minor writing errors: In Table S2, Table S3, and Table S5, words should not be split across lines; the “48h” label in the title of Table S3 is incorrect and should be corrected; duplicate “))” appears in line 201 at “FFA”; “Figs. 4C–D” should be plural.
12. The notation for statistical significance (P) should be unified in the main text and figure legends.
13. In the conclusion, “supramolecular eutectic platform (SPB)” is improperly labeled and should correspond to “Janus Eutectics.”

(Remarks on code availability)

Reviewer #2

(Remarks to the Author)
This manuscript reports an original and well-executed study on a supramolecular “Janus eutectic” system for transdermal delivery. The main novelty can be summarized as a carrier-free bioactive eutectic system integrating delivery and therapeutic functions within a single entity. The work is technically sound, visually compelling and supported by high-quality data throughout. The combination of multiscale simulations, ex vivo human skin experiments, in vitro biological assays and in vivo clinical evaluation provides a relatively comprehensive assessment of the system, extending from mechanistic insights to functional efficacy.

Overall, the study is interesting and potentially impactful, particularly for topical delivery and dermatological applications.

However, several methodological clarifications and conceptual limitations need to be addressed before the manuscript can be considered for publication in Nature Communications. I therefore recommend major revision.

1. Latin terms should be systematically italicized throughout the manuscript: stratum corneum, ex vivo, in vitro, etc.
2. Lines 81–82: statement regarding bisabolol penetration requires appropriate references.
3. The reproducibility of the study is currently limited by insufficient experimental details, especially regarding SPB preparation. SPB synthesis/formulation: The description in the main text and supp info is too brief. Please clarify: Nature of "mixing – line 108 and sheared at 300 rpm supp info p29": what devices (model, supplier) were used ? Under 10 MPa nitrogen: what apparatus + supplier?
4. Sonication: specify ultrasonic processor model and supplier ?
5. Temperature control: how was 333 K maintained?
6. The Franz cell experiments lack essential parameters: specify supplier, PBS receptor phase: specify pH ? Receptor chamber: specify volume ? Donor chamber conditions: was it occluded (with parafilm) or left open, specify ? Applied dose: "~100 µL sample" → confirm if identical volume for all formulations ? How itw as applied ?
7. Replicates: clarify number of biological replicates (human donors?) Skin source variability: number of donors ? age ? anatomical site?
8. SI-4.2 line 93 : sonicated ? more technical details (apparatus, frequency, ultrasound bath or cane ? model, supplier)
9. While the SPB system is described (albeit insufficiently), the "simple mixture" control is not adequately defined. The manuscript refers repeatedly to a simple mixture of D-panthenol, α-bisabolol and ethoxydiglycol, but: the preparation protocol is not clearly described in the main text nor supp info. It is unclear whether the mixture underwent:
the same thermal treatment, stirring conditions ? or sonication steps as SPB
10. Missing formulation details for the emulsion (Section 2.5) Clinical evaluation on anti-erythema efficacy introduces: "a facial emulsion containing SPB". However: No composition is provided, no formulation protocol is described, no physicochemical characteristics (O/W, viscosity, droplet size) are reported ?? The SPB concentration in the final formulation is not clearly stated ??
11. The use of Mexameter® MX18 methodology is reported in the results section only, however some missing information includes: measurement conditions (pressure, site, repetitions), environmental control (temperature, humidity), number of measurements per site. The clinical study compares: SPB emulsion vs water control. A more relevant control would be: the simple mixture formulation or at least the vehicle (emulsion) without SPB.
12. Statistics : normality tests were performed systematically ?
13. The distinction between "Janus eutectic" and existing NADES/DES/NES systems remains unclear, discuss more.

(Remarks on code availability)

Reviewer #3

(Remarks to the Author)
This manuscript describes the creation of a supramolecular Janus eutectic engineered from D-panthenol and 31 α-(-)-bisabolol (SPB). The authors claim that their "Janus Eutectics" concept is a breakthrough from traditional deep eutectic solvents that achieves three major advances: 1) bioactive components with complementary properties serve as building blocks, 2) the system integrates delivery or carrier and therapeutic capabilities, and 3) their system solves a reported efficacy-barrier-irritation paradox. The creation of the eutectic system requires the use of ethoxydiglycol, which is a skin penetration enhancer to stabilize the panthenol/bisabolol solution. The authors claim that their approach pioneers a generalizable strategy for reconstructing bioactive compounds through supramolecular engineering. They characterized their system using density functional theory, molecular dynamics simulations, and ex vivo tissue models. They also tested it on humans using water as a control. Although they report some enhancement in the properties of panthenol and bisabolol when part of SPB, these are moderate (Figs. 3B-C and Fig. 3E, and reduced the toxicity of bisabolol on the explant (though this viability measurement is not well defined)). Most important for publication in this journal, the generality of their approach is also not established, for example by utilizing another set of compounds with synergistic or complimentary properties that meet the same criteria of delivery and therapeutic enhancement reported. There are also a few concerns and specific issues that should be clarified:

1) The authors report the use of a control mixture, but it is not clear how this mixture of the three components is prepared or what process was particularly important to create the SPB. Was it the application of vacuum? If so, what are the details and what are the range of parameters that lead to the reported supramolecular network vs a simple mixture? Are there any temperature and reagent purity requirements for the synthesis of SPBs?

2) From this statement: "Similarly, as shown in Figs. 3D, both α-(-)-bisabolol alone and mixture of D-panthenol + α-(-)-bisabolol led to structural insult to the epidermis with equal 0.8% α-(-)-bisabolol dosage, indicated by abnormally organized basal cells, as well as hollow spaces in the upper epidermis." It seems the mixture is composed of only two components, whereas they state the mixture is 3 components elsewhere in the manuscript (e.g. line 250). Please clarify the definition of the experimental groups tested.

3) For the clinical evaluation of the SPBs, the authors did not include other control groups as they did for the in vitro and ex vivo studies (e.g. mixture, panthenol, bisabolol, etc). Furthermore, the protective effects that were observed for the SPB emulsion vs water were moderate. Given the moderate improvement over water and that no other reference test materials known to have this protective effect on skin were included, it is difficult to interpret the impact of the SPB.

(Remarks on code availability)

Version 1:

Reviewer comments:

Reviewer #1

(Remarks to the Author)
The authors have properly addressed all the questions raised by reviewers. It could be accepted for publication.

Reviewer #2

(Remarks to the Author)
The manuscript has been substantially improved following revision. The authors have adequately addressed all major concerns raised during the review process, resulting in a clearer and more robust study.

My only remaining reservation concerns the presentation of almost all figures. Several composite figures contain up to nine subpanels, making individual images difficult to examine without significant zooming. While this is manageable in the digital version, some details may become difficult or impossible to interpret in a standard A4 printed format. The authors may wish to consider enlarging key panels or redistributing the figures to improve readability.

Reviewer #3

(Remarks to the Author)
The revised manuscript is improved and the additional pilot clinical study answers questions regarding the control test material used to show superiority. There seems to be some improvement from their system relative to water and the "mixture" control. However, questions remain regarding the generalization of the results, and per the reviewer's response, the innovation of the work to justify publication in this journal. The authos state: "This principle has already been most maturely applied in pharmaceutical cocrystals, which share the same conceptual basis. However, a major current challenge is the lack of suitable characterization methods to verify the impact of complex formulation matrices on supramolecular structure. To address this, we have provided a comparative illustration of the two formulations in terms of efficacy and soothing performance, and we hope this adequately clarifies the issue."
From this response the authors state that the work addresses a characterization problem or challenge. How is this consistent with the narrative in the manuscript regarding the rationale for the janus system? To support generalization of a concept, they should provide examples using systems with other componens, where the results also apply. If the approach is not generalizable, those statements should be removed, amended, or the authors should provide evidence to support their conclusion.

Open Access This Peer Review File is licensed under a Creative Commons Attribution 4.0 International License, which permits use, sharing, adaptation, distribution and reproduction in any medium or format, as long as you give appropriate credit to the original author(s) and the source, provide a link to the Creative Commons license, and indicate if changes were made.
In cases where reviewers are anonymous, credit should be given to 'Anonymous Referee' and the source.
The images or other third party material in this Peer Review File are included in the article's Creative Commons license, unless indicated otherwise in a credit line to the material. If material is not included in the article's Creative Commons license and your intended use is not permitted by statutory regulation or exceeds the permitted use, you will need to obtain permission directly from the copyright holder.
To view a copy of this license, visit https://creativecommons.org/licenses/by/4.0/

June 2, 2026

Dear Reviewers,

Re: Manuscript ID: NCOMMS-26-005126A

Title: “Supramolecular Janus Eutectic Engineered from Panthenol and Bisabolol Overcomes Transdermal Delivery Paradoxes”

We would like to express our sincere gratitude to you for the time and effort spent in reviewing our manuscript. We greatly appreciate the insightful and constructive comments provided. These suggestions have been invaluable in helping us improve the quality and clarity of our work.

We have carefully considered all the comments and revised the manuscript accordingly. In the revised manuscript, all changes are highlighted using the [Track Changes / Red Highlighting] function for ease of identification.

Below, we provide a detailed, point-by-point response to each comment. The reviewer’s comments are presented in ***bold italic***, followed by our responses in plain text.

Thank you again for your consideration of our manuscript.

Sincerely,

Jiaheng ZHANG

Sauvage Laboratory for Smart Materials, School of Materials Science and Engineering, Harbin Institute of Technology (Shenzhen), Shenzhen 518055, China.

E-mail: zhangjiaheng@hit.edu.cn

RESPONSES TO REVIEWER #1

This study provides a detailed analysis of the advantages and underlying mechanisms of the SPB through a combination of various experimental models and corresponding theoretical calculations. The experimental design is relatively rigorous and closely aligned with practical application scenarios, demonstrating considerable academic merit and promising translational potential. However, several issues remain that require further refinement and clarification.

Response: We sincerely thank the Reviewer for this thoughtful and encouraging summary of our study. We greatly appreciate the recognition of the rigorous experimental design, the close alignment with practical application scenarios, and the acknowledgment of the academic merit and translational potential of this work.

We fully agree that several aspects required further refinement and clarification. We have carefully addressed all the specific issues raised by the Reviewer in the revised manuscript. The detailed, point-by-point responses are provided below. We believe these revisions have further improved the clarity and overall quality of the manuscript, and we hope the revised version now meets the Reviewer's expectations.

1. The introduction mentions drawbacks of traditional delivery systems, such as the complexity of preparation, toxicity, and stability issues of nanocarriers. Relevant reports should be added to provide theoretical support.

Response: We thank you for pointing out this neglected reference support. We have now incorporated additional references (Refs. 7–9) addressing the preparation process, toxicity, and stability, as indicated on lines 461–468.

[7]. Mehta, M. et al. Lipid-Based Nanoparticles for Drug/Gene Delivery: An Overview of the Production Techniques and Difficulties Encountered in Their Industrial Development. ACS Mater Au 3, 600-619 (2023).

[8]. Edo, G.I. et al. Toxicity of nanostructures in drug delivery applications. Pharm Sci Adv 4, 100117 (2026).

[9]. Haghighi, E. et al. Navigating the intricate in-vivo journey of lipid nanoparticles tailored for the targeted delivery of RNA therapeutics: a quality-by-design approach. J Nanobiotechnology 22, 710 (2024).

2. The term “Janus Eutectics” is used in the text; its definition or distinction from DES should be clarified. Additionally, it is recommended to modify expressions like “breakthrough” and

"revolutionary" to more reasonable alternatives.

Response: We agree with the reviewer that this is an important point. We have revised the text in the Introduction section, as indicated on lines 61–71 now reads:

Deep Eutectic Solvents (DES) are characterized by a melting point significantly lower than that of any individual component[12]. As most components are solids with similar physicochemical properties, high viscosity is also a notable feature of DES, which constitutes a core bottleneck limiting its full functional potential and industrial application. Nevertheless, the good biocompatibility and synergistic functions of DES still offer considerable application value, with Natural Deep Eutectic Solvents (NADES) being particularly favored[13]. Based on DES, we propose the concept of "JANUS Eutectics", emphasizing that the precursors are composed of components with substantially different or even opposite properties (e.g., hydrophilic vs. lipophilic) yet functionally complementary. The supramolecular structure is maintained by hydrogen bonding, with the glass transition temperature (Tg) as the core characteristic, and exhibits the following properties: (1) bioactive components serve as building blocks with unified yet complementary properties, (2) inherent integration of delivery and therapeutic functions, and (3) resolution of the historical efficacy-barrier-irritation paradox.

3. The source of logP data and its physical meaning should be explained to ensure logical rigor.

Response: We agree with the reviewer that this is an important point. We have revised the text in the Introduction section, as indicated on lines 83–87 now reads:

The logarithm of the partition coefficient (Log P) is a quantitative measure of compound lipophilicity and can be calculated using various models[20], such as SwissADME. Log P is commonly used to preliminarily assess the ease with which an active agent penetrates the skin lipid barrier. It exhibits an inverted U-shaped parabolic relationship with permeation efficiency (Hansch model), with the optimal range being 1–4.

4. The rationale for selecting a 1:1:1 molar ratio for the SPB should be provided. It should also be clarified whether other ratios were attempted and how this specific ratio was optimized.

Response: We agree with the reviewer that this is an important point. Please allow me to explain: For classical DES or eutectic systems formed through hydrogen bonding, the component ratio is always 1:1 molar ratio. This is both empirically derived and necessary to ensure accurate allocation of hydrogen bonding sites, thereby guaranteeing that the actual structure matches that predicted by simulation calculations and characterization analyses. Furthermore, a high proportion of panthenol

would result in poor fluidity of the system, which is unfavorable for its production and application.

5. The IGMH analysis needs further elaboration to ensure consistency with hydrogen bond sites and characterization data. Supporting evidence for using ESP distribution to predict amphiphilicity should be supplemented.

Response: Thank you for this suggestion. We have revised the text in the 2.1 section, as indicated on lines128–132 now reads: It can be clearly observed that the amino group of D-panthenol and the hydroxyl group of α-(-)-bisabolol form strong hydrogen bonds, while the ether bond near the hydroxyl group of ethoxydiglycol forms hydrogen bonds with the hydroxyl group of D-panthenol. Interactions at other sites are predominantly van der Waals forces, corresponding to **Fig. 1A**.

We have now incorporated additional references (Refs. 36-37) supporting ESP can be used to analyze amphiphilicity, as indicated on lines 506–509.

[36]. Murray, J.S. & Politzer, P. Molecular electrostatic potentials and noncovalent interactions. *Wiley Interdisciplinary Reviews: Computational Molecular Science* **7** (2017).

[37]. Murray, J.S., Brinck, T., Lane, P., Paulsen, K. & Politzer, P. Statistically-based interaction indices derived from molecular surface electrostatic potentials: a general interaction properties function (GIPF). *Journal of Molecular Structure: THEOCHEM* **307**, 55-64 (1994).

6. The experimental design of the stress test should be detailed, including the specific amount used in the test.

Response: We agree with the reviewer that this is an important point. We have supplemented the text in the supporting information, as indicated on lines 81–89 now reads:

pH stability test: 0.2% SPB or physical mixture aqueous solutions were adjusted to pH gradients using glycolic acid or KOH (Macklin, Shanghai, China). After 48 hr at room temperature (RT), the contents of D-panthenol and α-(-)-bisabolol were determined.

Temperature stability test: 0.2% SPB aqueous solutions were stored at 4°C, RT, and 50°C. The contents of D-panthenol and α-(-)-bisabolol were measured on days 0, 5, 10, and 30.

Oxidant stability test: 0.2% SPB aqueous solution was mixed with hydrogen peroxide at a mass ratio of 95:5. After 48 hr, the contents of D-panthenol and α-(-)-bisabolol were determined.

7. Figure issues: The numbers in Figure 1C and Figure 2B are not clear enough.

Response: We agree with the reviewer that this is an important point. Figures 1 and 2 have been

optimized and replaced, as indicated on lines 177–178 and lines 244–245.

8. The scientific validity and accuracy of the SkinEthic™ RHE model used in the study should be supported with references.

Response: Thank you for this suggestion. We have now incorporated additional references (Refs. 43–44) addressing the preparation process, toxicity, and stability, as indicated on lines 543–547.

[43]. Catarino, C.M. et al. Skin corrosion test: a comparison between reconstructed human epidermis and full thickness skin models. *European Journal of Pharmaceutics and Biopharmaceutics* **125**, 51-57 (2018).

[44]. Liu, Y. et al. A Supramolecular Salicylic Acid-Betaine with Superior Epidermal Benefits and Better Tolerance: In vitro Proof of Concept Studies. *Skin Pharmacology and Physiology* (2026).

9. The rationale for selecting a 2% concentration in the RHE model tests should be provided.

Response: We agree with the reviewer that this is an important point. We have supplemented the text in the in the 2.3 section, as indicated on lines 260–264 now reads:

This dose was selected based on previous data suggesting 0.8% α-(-)-bisabolol could induce stress response in the RHE model, while 0.5% was often not (data not published before). Thus in order to assess improved margin of tolerance with SPB, 2% dose containing 0.8% α-(-)-bisabolol was used.

10. The basis for choosing the two concentrations in the TRPV1 inhibition rate experiment should be explained.

Response: We agree with the reviewer that this is an important point. We have supplemented the text in the supporting information, as indicated on lines 162–165 now reads:

intended to demonstrate any potential dose-dependency in benefits. As bisabolol was tolerated well at 40 μM but not at 50 μM[11], a dosage of 42 μM (9.4 μg/mL) was used for high dose, and half of that as low dose (4.7 μg/mL).

[11].Tan, C. et al. The combination of allantoin, bisabolol, D-panthenol and dipotassium glycyrrhizinate mitigates UVB-induced PGE2 synthesis by keratinocytes. *International Journal of Cosmetic Science* **46**, 691-701 (2024).

11. Minor writing errors: In Table S2, Table S3, and Table S5, words should not be split across lines; the "48h" label in the title of Table S3 is incorrect and should be corrected; duplicate "))" appears in line 201 at "FFA"; "Figs. 4C–D" should be plural.

Response: Thank you for your careful observation; we greatly appreciate it. The above content has now been corrected, as indicated on lines 221, etc.

12. The notation for statistical significance (P) should be unified in the main text and figure legends.

Response: Thank you for your careful correction; we greatly appreciate it. The entire manuscript has now been reviewed and corrected.

13. In the conclusion, "supramolecular eutectic platform (SPB)" is improperly labeled and should correspond to "Janus Eutectics."

Response: Thank you for your careful observation; we greatly appreciate it. We have revised the text in the conclusion section, as indicated on lines 418–419.

RESPONSES TO REVIEWER #2

This manuscript reports an original and well-executed study on a supramolecular "Janus eutectic" system for transdermal delivery. The main novelty can be summarized as a carrier-free bioactive eutectic system integrating delivery and therapeutic functions within a single entity. The work is technically sound, visually compelling and supported by high-quality data throughout. The combination of multiscale simulations, ex vivo human skin experiments, in vitro biological assays and in vivo clinical evaluation provides a relatively comprehensive assessment of the system, extending from mechanistic insights to functional efficacy.

Overall, the study is interesting and potentially impactful, particularly for topical delivery and dermatological applications. However, several methodological clarifications and conceptual limitations need to be addressed before the manuscript can be considered for publication in Nature Communications. I therefore recommend major revision.

Response: We sincerely thank the Reviewer for this thorough, thoughtful, and highly encouraging assessment of our work. We greatly appreciate the recognition of the study's novelty, specifically the concept of a carrier-free bioactive eutectic system that integrates delivery and therapeutic functions. We are also grateful for the positive comments on the technical rigor, the high-quality data, and the comprehensive combination of multiscale simulations, ex vivo experiments, in vitro assays, and in vivo clinical evaluation.

We fully acknowledge the Reviewer's concern regarding the need for methodological clarifications and the discussion of conceptual limitations. We have taken these points very seriously and have addressed each of them in the revised manuscript as detailed in our point-by-point responses below.

We believe these revisions have significantly strengthened the manuscript, and we hope that the revised version now meets the standards for publication in *Nature Communications*.

1. Latin terms should be systematically italicized throughout the manuscript: stratum corneum, ex vivo, in vitro, etc.

Response: Thank you for your careful correction; we greatly appreciate it. The entire manuscript has now been reviewed and corrected, as indicated on lines 89, 97, 111, 185, 220, 283, and 421.

2. Lines 81–82: statement regarding bisabolol penetration requires appropriate references.

Response: We thank you for pointing out this neglected reference support. We have now incorporated additional references (Refs. 27–28) supporting bisabolol assists penetration, as indicated on lines 506–509.

[27]. Kadir, R. & Barry, B.W. α-Bisabolol, a possible safe penetration enhancer for dermal and transdermal therapeutics. *International Journal of Pharmaceutics* **70**, 87-94 (1991).

[28]. Cui, Y. et al. Enhancement and mechanism of transdermal absorption of terpene-induced propranolol hydrochloride. *Arch Pharm Res* **34**, 1477-1485 (2011).

3. The reproducibility of the study is currently limited by insufficient experimental details, especially regarding SPB preparation. SPB synthesis/formulation: The description in the main text and supp info is too brief. Please clarify: Nature of "mixing – line 108 and sheared at 300 rpm supp info p29": what devices (model, supplier) were used ? Under 10 MPa nitrogen: what apparatus + supplier?

Response: We thank you for pointing out these neglected experimental details. We have supplemented the text in the supporting information, as indicated on lines 28–41 now reads:

The D-Panthenol (purity >98%; Hangzhou Xinfu, Zhejiang, China) and α-(-)-bisabolol (purity >99%; Givaudan) were in a 1:1 molar ratio were heated in a water bath at 333 K on a heated magnetic stirrer (IKA RCT basic) under nitrogen protection (10 L cylinder, high-purity nitrogen, Shenzhen Shenter Industrial Gas Co., Ltd., China) with stirring at 300 rpm for 6 h until no phase separation occurred. The beaker was then placed in an ultrasonic cell crusher (SCIENTZ-IID, Ningbo Xinzhi, China); a No. 6 horn (probe) was immersed to half the depth of the liquid, centered, and ultrasonication was performed at 300 W for 1 h (accompanied by an ice-water bath) with an on/off cycle of 2 s on / 2 s off. Then an equimolar amount of ethoxydiglycol (Gattefossé SAS) was added, and the above process was repeated to obtain a resultant supramolecular D-panthenol-α-(-)-bisabolol Janus eutectic (SPB) as a pale yellow, clear, transparent liquid.

4. Sonication: specify ultrasonic processor model and supplier ?

Response: We thank you for pointing out these neglected experimental details. We have supplemented the text in the supporting information, as indicated on lines 32–33 now reads:

The beaker was then placed in an ultrasonic cell crusher (SCIENTZ-IID, Ningbo Xinzhi, China).

5. Temperature control: how was 333 K maintained?

Response: We thank you for pointing out these neglected experimental details. The heated magnetic stirrer has built-in temperature control; water-bath heating helps maintain the temperature.

6. The Franz cell experiments lack essential parameters: specify supplier, PBS receptor phase: specify pH ? Receptor chamber: specify volume ? Donor chamber conditions: was it occluded

(with parafilm) or left open, specify ? Applied dose: "~100 μL sample" → confirm if identical volume for all formulations ? How itw as applied ?

Response: We thank you for pointing out these neglected experimental details. Supplier information is included in the supplement file L92-93. We have supplemented the text in the supporting information, as indicated on lines 100–106 now reads:

For PBS receptor phase was 0.1M with pH of 7.4. It was non-occluded after each treatment was applied topically at a volume of 100 μL followed with glass stick smearing. The apparatus was maintained at a constant temperature of 32 degree Celsius.

7. Replicates: clarify number of biological replicates (human donors?) Skin source variability: number of donors ? age ? anatomical site?

Response: We thank you for pointing out these neglected experimental details. We have supplemented the text in the supporting information, as indicated on lines 94–99 now reads:

Adult female abdominal skin discards from liposuction procedure were employed for the penetration study. A total of three donors were included, with one replicate per treatment from each donor. Due to privacy considerations, donor age was not disclosed. In order to control the quality of skin explants, trans-epidermal water loss (TEWL) after conditioning in the Franz cell system was measured to ensure integrity of the skin sheets, with an upper limit set at 15 $g/m^2/hr$.

8. SI-4.2 line 93 : sonicated ? more technical details (apparatus, frequency, ultrasound bath or cane ? model, supplier)

Thanks for raising this point. Corresponding clarification has been made in the 5.2 session in supplement file L121-122, as "using a KQ3200E ultrasonic cleaner (40KHz, Kunshan Shumei, CN) at 150W for 60 mins".

9. While the SPB system is described (albeit insufficiently), the "simple mixture" control is not adequately defined. The manuscript refers repeatedly to a simple mixture of D-panthenol, α-bisabolol and ethoxydiglycol, but: the preparation protocol is not clearly described in the main text nor supp info. It is unclear whether the mixture underwent: the same thermal treatment, stirring conditions ? or sonication steps as SPB

Response: We thank you for pointing out these neglected experimental details. We have supplemented the text in the supporting information, as indicated on lines 42–44 now reads:

Simple mixture: D-Panthenol, α-(-)-bisabolol, and ethoxydiglycol were mixed at a 1:1:1 molar ratio at

room temperature (RT), stirred briefly with a glass rod without any instrumental assistance, yielding a colorless, clear, transparent liquid.

10. Missing formulation details for the emulsion (Section 2.5) Clinical evaluation on anti-erythema efficacy introduces: "a facial emulsion containing SPB". However: No composition is provided, no formulation protocol is described, no physicochemical characteristics (O/W, viscosity, droplet size) are reported ?? The SPB concentration in the final formulation is not clearly stated ??

We fully appreciate this comment and agree that the formulation protocol and composition are important for understanding the clinical advantages of SPB. Due to the confidentiality of the formulation, and to still address this point, we conducted additional clinical tests using a base emulsion containing only the supramolecular components as the sole active ingredient, with a physical mixture group as the control. The formulation details are provided in Section 2.5 (L361-367) and the Supporting Information (Table S6, L266-267).

To compare the performance of SPB and a simple mixture in a formulation, a base emulsion containing only 2% of the supramolecular component as the sole active ingredient (Table S6) was used in an arm patch test under Sodium Dodecyl Sulfate (SDS) stimulation to evaluate its soothing properties. The viscosities (Brookfield, DV2T: 4#50r, 1924 cp, 18.1%; 1912 cp, 47.8%) and pH (5.9 and 5.7) of the two emulsions (SPB group and simple mixture group) were very similar, with droplet sizes of 93,893 nm and 36,037 nm (n=3), respectively.

Table S6. Composition of formula components

**SPB**		**Mix**		**/**
**Compents**	**Contents (%)**	**Compents**	**Contents (%)**	**Category**
SPB	*2*	*PANTHENOL*	*0.72*	*ACTIVE*
		BISABOLOL	*0.8*	
		Ethoxydiglycol	*0.48*	
SQUALANE	*2*	*SQUALANE*	*2*	*EMOLLIENT*
Caprylic/Capric Triglyceride	*5*	*Caprylic/Capric Triglyceride*	*5*	*EMOLLIENT*
STEARIC ACID	*0.5*	*STEARIC ACID*	*0.5*	*SURFACTANT*
GLYCERYL STEARATE SE	*0.8*	*GLYCERYL STEARATE SE*	*0.8*	*SURFACTANT*

XANTHAN GUM	*0.1*	*XANTHAN GUM*	*0.1*	*POLYMER*
AMMONIUM POLYACRYLOYLDIMETHYL TAURATEPHENOXYETHANOL	*0.4*	*AMMONIUM POLYACRYLOYLDIMETHYL TAURATEPHENOXYETHANOL*	*0.4*	*POLYMER*
PHENOXYETHANOL	*0.2*	*PHENOXYETHANOL*	*0.2*	*Preservative*
GLYCERIN	*3*	*GLYCERIN*	*3*	*SOLVENT*
PENTYLENE GLYCOL	*1.5*	*PENTYLENE GLYCOL*	*1.5*	*SOLVENT*
CITRIC ACID	*QS*	*CITRIC ACID*	*QS*	*pH adjustment*
WATER	*QS*	*WATER*	*QS*	*SOLVENT*

11. The use of Mexameter® MX18 methodology is reported in the results section only, however some missing information includes: measurement conditions (pressure, site, repetitions), environmental control (temperature, humidity), number of measurements per site. The clinical study compares: SPB emulsion vs water control. A more relevant control would be: the simple mixture formulation or at least the vehicle (emulsion) without SPB.

We fully agree with this comment, as the present study only demonstrates the effectiveness of the SPB containing emulsion, rather than active itself. Water was chosen as benchmark as it was shown that water application provides instant refreshing effect against erythema, etc. (https://doi.org/10.1155/dth/3695790). We aim to conduct proof of mechanism and superiority study in higher number of subjects in the coming years, focusing on subjects with vulnerable skin. We agree that it is important not only to examine SPB's performance in the formulation but also to demonstrate its difference from a simple mixture. Accordingly, we have included an additional 31-subject clinical trial, which shows that SPB holds greater application potential than the simple mixture. As indicated on lines 367–383 now reads:

This study was approved by the local ethics committee (approval No. BXLL2026016). After obtaining informed consent, 31 self-reported sensitive-skinned Chinese volunteers (mean age 34 yr) were recruited. The emulsion was applied twice daily for three consecutive days, followed by induction of skin redness with SDS, and then the emulsion was reapplied to record skin recovery. The BC group refers to no application after induction, and untreated means no treatment. Transepidermal water loss (TEWL), erythema index, and images were measured using a TEWL meter (SWL5201, Delfin) and an Antera 3D imager (HC1023Q, Delfin) at T0 (immediately after SDS induction), T1 (immediately after product application), T30 min (30 mins after product application), and T2H (2 hours after product application).

As shown in **Fig. 5A** and **5C**, both SPB and the simple mixture reduced TEWL and repaired the skin barrier, with SPB showing significantly better effects, both immediately and within two hours. Similar results were observed for the erythema index (**Fig. 5B, 5D**); SPB exhibited a more pronounced anti-redness and repair effect than the simple mixture, with representative images shown in **Fig. S11**. This indicates that preparing supramolecular JANUS eutectics from components with potentially complementary functions in a formulation is a highly promising strategy for benefit enhancement.

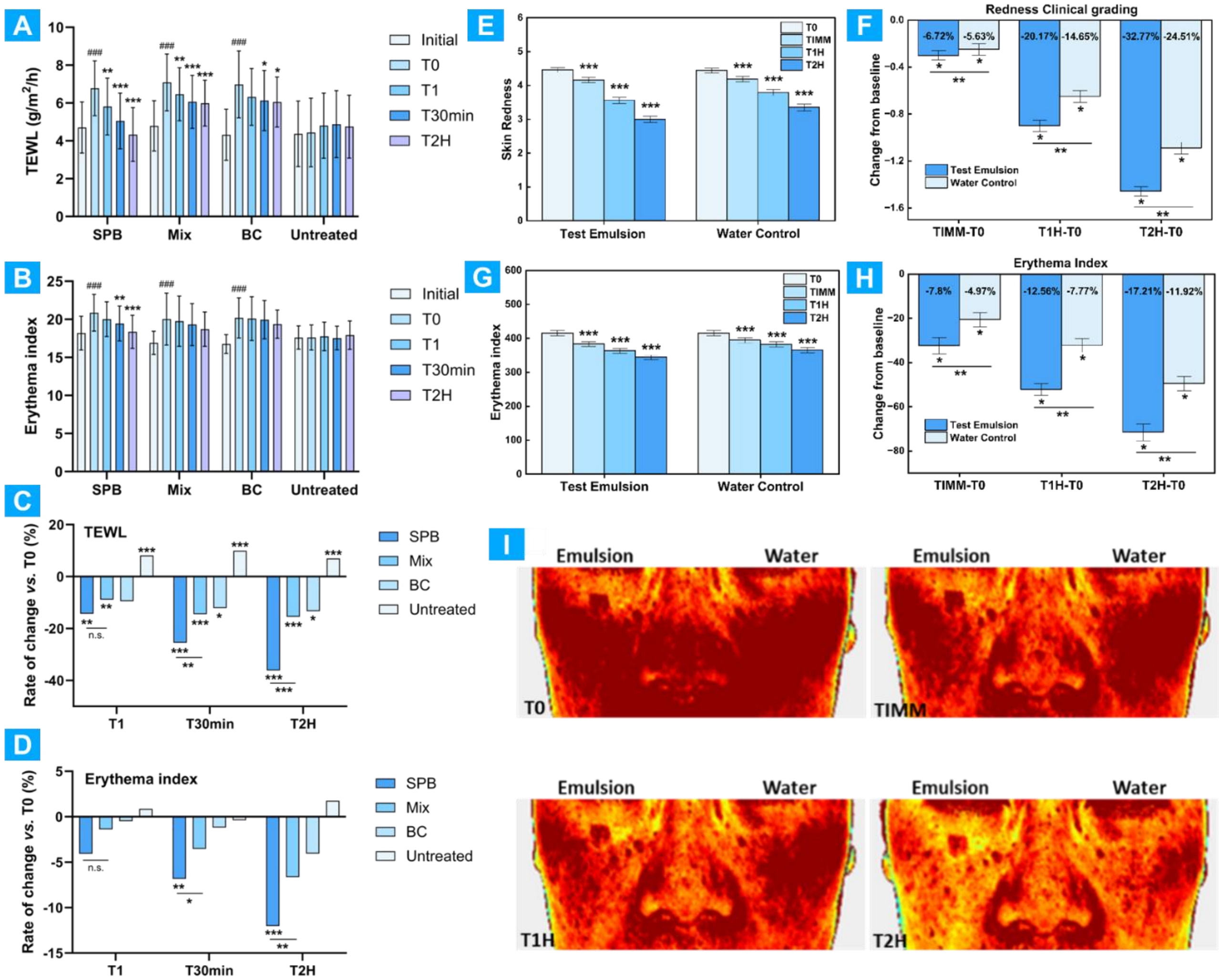

Fig. 5 Clinical anti-redness performance of emulsion containing SPB. (A, C) Pre- and post-treatment arm TEWL changes measured by TEWL meter. (B, D) Pre- and post-treatment arm erythema index changes measured by Antera 3D imager. (### *P*-value < 0.001 versus initial, * *P*-value < 0.05, ** *P*-value < 0.01, and *** *P*-value < 0.001). (E-F) Change from baseline in dermatologist assessment score of skin redness. (G-H) Change from baseline in erythema index measured by Mexameter® MX 18. (* *P*-value < 0.05 and *** *P*-value < 0.001 versus baseline, ** *P*-value < 0.01 between emulsion and water control). (I) Representative images taken by Tivi700 of subject at T0, TIMM, T1H and T2H.

The operating procedure for the Mexameter® MX18, which is critical for the scientific rigor and reproducibility of the experiment, has been added to the Supporting Information, as indicated on lines 175–187 now reads:

9. Mexameter® MX18 methodology

Measurement conditions (pressure, site, repetitions): The manufacturer's instructions for the Mexameter® MX18 do not specify a quantitative pressure value. However, the spring-loaded probe head is designed to support consistent contact pressure on the skin. To ensure measurement reproducibility in practice, a standardized operating procedure was followed, whereby the probe was positioned perpendicular to the skin surface and applied with a firm, fast, and controlled movement to ensure full contact of the measurement surface with the skin. Triplicated Mexameter measurements was taken on the center of each subject's right or left cheek (at the intersection of lines extending down from the outer corner of the eye and horizontally across the bottom of the nose) on the opposite side of face as Vapometer measurements.

Environmental control (temperature, humidity): According to record from whole clinical period : Temperature: 21.2 ± 0.9°C; Humidity: 51.4 ± 2.3 %

12. Statistics : normality tests were performed systematically ?

Thanks for raising this point. Corresponding clarification has been made in the statistical session in supplement file L202–207, as " For numerical parameters, normality and equal variance were first verified by Shapiro-Wilk and F-test first, then subjected to either Analysis of variance (ANOVA) or Student's t-test. Post-hoc comparisons were conducted with Dunnett or Tukey adjustments depending on comparison framing. Statistically significant differences were highlighted by asterisks (* $p<0.05$, ** $p<0.01$, *** $p<0.001$). Such statistical analyses and diagram making was conducted using GraphPad Prism software."

13. The distinction between "Janus eutectic" and existing NADES/DES/NES systems remains unclear, discuss more.

Response: We agree with the reviewer that this is an important point. We have revised the text in the Introduction section, as indicated on lines 61–71 now reads:

Deep Eutectic Solvents (DES) are characterized by a melting point significantly lower than that of any individual component[12]. As most components are solids with similar physicochemical properties, high viscosity is also a notable feature of DES, which constitutes a core bottleneck limiting its full functional potential and industrial application. Nevertheless, the good biocompatibility and synergistic functions

of DES still offer considerable application value, with Natural Deep Eutectic Solvents (NADES) being particularly favored[13]. Based on DES, we propose the concept of “JANUS Eutectics”, emphasizing that the precursors are composed of components with substantially different or even opposite properties (e.g., hydrophilic vs. lipophilic) yet functionally complementary. The supramolecular structure is maintained by hydrogen bonding, with the glass transition temperature (Tg) as the core characteristic, and exhibits the following properties: (1) bioactive components serve as building blocks with unified yet complementary properties, (2) inherent integration of delivery and therapeutic functions, and (3) resolution of the historical efficacy-barrier-irritation paradox.

RESPONSES TO REVIEWER #3

This manuscript describes the creation of a supramolecular Janus eutectic engineered from D-panthenol and 31 α-(-)-bisabolol (SPB). The authors claim that their "Janus Eutectics" concept is a breakthrough from traditional deep eutectic solvents that achieves three major advances: 1) bioactive components with complementary properties serve as building blocks, 2) the system integrates delivery or carrier and therapeutic capabilities, and 3) their system solves a reported efficacy-barrier-irritation paradox. The creation of the eutectic system requires the use of ethoxydiglycol, which is a skin penetration enhancer to stabilize the panthenol/bisabolol solution. The authors claim that their approach pioneers a generalizable strategy for reconstructing bioactive compounds through supramolecular engineering. They characterized their system using density functional theory, molecular dynamics simulations, and ex vivo tissue models. They also tested it on humans using water as a control. Although they report some enhancement in the properties of panthenol and bisabolol when part of SPB, these are moderate (Figs. 3B-C and Fig. 3E, and reduced the toxicity of bisabolol on the explant (though this viability measurement is not well defined)). Most important for publication in this journal, the generality of their approach is also not established, for example by utilizing another set of compounds with synergistic or complimentary properties that meet the same criteria of delivery and therapeutic enhancement reported. There are also a few concerns and specific issues that should be clarified:

Response: We sincerely thank the Reviewer for this careful and critical assessment of our work. We greatly appreciate the recognition of the Janus Eutectics concept and the detailed comments that have guided us in improving the manuscript.

Regarding the moderate enhancement observed in some properties, we respectfully note that even modest improvements in transdermal delivery and irritation reduction can be therapeutically meaningful, particularly in a carrier-free integrated system.

Regarding the cell viability assay, the SkinEthic™ RHE model employed in this study represents one of the most well-established new approach methodologies currently available. It offers clearly defined endpoints, high accuracy, and excellent reproducibility. Its highly biomimetic architecture and barrier function closely resemble those of native human epidermis, enabling it to simulate the penetration, barrier, and reactivity responses of human skin to exogenous substances. It provides a human biology-based, ethically sound, and scientifically valid alternative to animal testing. The SkinEthic™ RHE assay has been adopted by the Organisation for Economic Co-operation and Development (OECD) and is formally included in the test guidelines for skin irritation and corrosion, namely OECD

TG 439 (skin irritation) and OECD TG 431 (skin corrosion). It is also endorsed by the Chinese national standard GB/T 6886.23 (biological evaluation of medical devices—irritation test) and the international medical device standard ISO 10993-23. We hope this clarification adequately addresses the concern.

Regarding the generality of the approach, its chemical foundation lies in the predictable and semi-rational design of supramolecular structures to achieve synergistic effects—such as improved physicochemical properties (particularly solubility and permeability), dual pharmacodynamic synergy, and reduced toxicity and side effects. This principle has already been most maturely applied in pharmaceutical cocrystals, which share the same conceptual basis. However, a major current challenge is the lack of suitable characterization methods to verify the impact of complex formulation matrices on supramolecular structure. To address this, we have provided a comparative illustration of the two formulations in terms of efficacy and soothing performance, and we hope this adequately clarifies the issue.

All specific issues raised by the Reviewer are addressed point-by-point below. We believe these revisions and clarifications have substantially strengthened the manuscript, and we hope it is now suitable for publication in *Nature Communications*.

1) The authors report the use of a control mixture, but it is not clear how this mixture of the three components is prepared or what process was particularly important to create the SPB. Was it the application of vacuum? If so, what are the details and what are the range of parameters that lead to the reported supramolecular network vs a simple mixture? Are there any temperature and reagent purity requirements for the synthesis of SPBs?

Response: We thank you for pointing out these neglected experimental details. As you noted, the key difference between SPB and a simple mixture lies in whether the molecules achieve sufficient integration under specific conditions of temperature, pressure, ultrasound, and protective gas (or vacuum). We have supplemented the text in the supporting information, as indicated on lines 28–44 now reads:

The D-Panthenol (purity >98%; Hangzhou Xinfu, Zhejiang, China) and α-(-)-bisabolol (purity >99%; Givaudan) were in a 1:1 molar ratio were heated in a water bath at 333 K on a heated magnetic stirrer (IKA RCT basic) under nitrogen protection (10 L cylinder, high-purity nitrogen, Shenzhen Shenter Industrial Gas Co., Ltd., China) with stirring at 300 rpm for 6 h until no phase separation occurred. The beaker was then placed in an ultrasonic cell crusher (SCIENTZ-IID, Ningbo Xinzhi, China); a No. 6 horn (probe) was immersed to half the depth of the liquid, centered, and ultrasonication was

performed at 300 W for 1 h (accompanied by an ice-water bath) with an on/off cycle of 2 s on / 2 s off. Then an equimolar amount of ethoxydiglycol (Gattefossé SAS) was added, and the above process was repeated to obtain a resultant supramolecular D-panthenol-α-(-)-bisabolol Janus eutectic (SPB) as a pale yellow, clear, transparent liquid.

Simple mixture: D-Panthenol, α-(-)-bisabolol, and ethoxydiglycol were mixed at a 1:1:1 molar ratio at room temperature (RT), stirred briefly with a glass rod without any instrumental assistance, yielding a colorless, clear, transparent liquid.

2) From this statement: "Similarly, as shown in Figs. 3D, both α-(-)-bisabolol alone and mixture of D-panthenol + α-(-)-bisabolol led to structural insult to the epidermis with equal 0.8% α-(-)-bisabolol dosage, indicated by abnormally organized basal cells, as well as hollow spaces in the upper epidermis." It seems the mixture is composed of only two components, whereas they state the mixture is 3 components elsewhere in the manuscript (e.g. line 250). Please clarify the definition of the experimental groups tested.

Response: Thank you very much for pointing out this typo. The mixtures were all simply prepared by mixing the three components in the same ratio. We have corrected it at the relevant location, as shown on line 280.

Similarly, as shown in **Figs. 3D**, both α-(-)-bisabolol alone and mixture of D-panthenol, α-(-)-bisabolol and ethoxydiglycol led to structural insult to the epidermis with equal 0.8% α-(-)-bisabolol dosage, indicated by abnormally organized basal cells, as well as hollow spaces in the upper epidermis.

3) For the clinical evaluation of the SPBs, the authors did not include other control groups as they did for the in vitro and ex vivo studies (e.g. mixture, panthenol, bisabolol, etc). Furthermore, the protective effects that were observed for the SPB emulsion vs water were moderate. Given the moderate improvement over water and that no other reference test materials known to have this protective effect on skin were included, it is difficult to interpret the impact of the SPB.

We fully agree with this comment, as the present study only demonstrates the effectiveness of the SPB containing emulsion, rather than active itself. Water was chosen as benchmark as it was shown that water application provides instant refreshing effect against erythema, etc. (https://doi.org/10.1155/dth/3695790). We aim to conduct proof of mechanism and superiority study in higher number of subjects in the coming years, focusing on subjects with vulnerable skin. We agree that it is important not only to examine SPB's performance in the formulation but also to demonstrate its difference from a simple mixture. Accordingly, we have included an additional 31-subject clinical

trial, which shows that SPB holds greater application potential than the simple mixture. As indicated on lines 367–383 now reads:

This study was approved by the local ethics committee (approval No. BXLL2026016). After obtaining informed consent, 31 self-reported sensitive-skinned Chinese volunteers (mean age 34 yr) were recruited. The emulsion was applied twice daily for three consecutive days, followed by induction of skin redness with SDS, and then the emulsion was reapplied to record skin recovery. The BC group refers to no application after induction, and untreated means no treatment. Transepidermal water loss (TEWL), erythema index, and images were measured using a TEWL meter (SWL5201, Delfin) and an Antera 3D imager (HC1023Q, Delfin) at T0 (immediately after SDS induction), T1 (immediately after product application), T30 min (30 mins after product application), and T2H (2 hours after product application).

As shown in Fig. 5A and 5C, both SPB and the simple mixture reduced TEWL and repaired the skin barrier, with SPB showing significantly better effects, both immediately and within two hours. Similar results were observed for the erythema index (Fig. 5B, 5D); SPB exhibited a more pronounced anti-redness and repair effect than the simple mixture, with representative images shown in Fig. S11. This indicates that preparing supramolecular JANUS eutectics from components with potentially complementary functions in a formulation is a highly promising strategy for benefit enhancement.

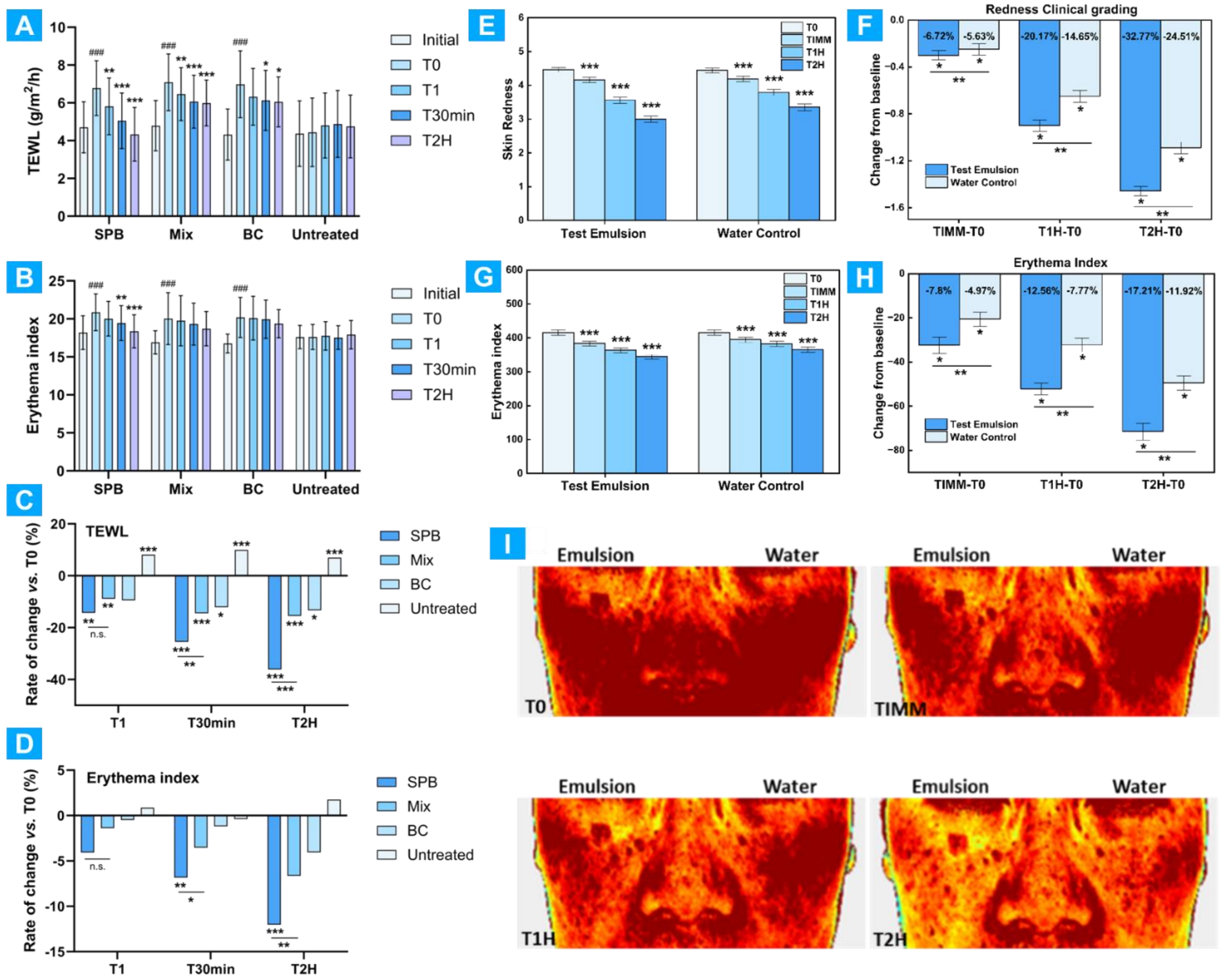

Fig. 5 Clinical anti-redness performance of emulsion containing SPB. (A, C) Pre- and post-treatment arm TEWL changes measured by TEWL meter. (B, D) Pre- and post-treatment arm erythema index changes measured by Antera 3D imager. (### *P*-value < 0.001 versus initial, * *P*-value < 0.05, ** *P*-value < 0.01, and *** *P*-value < 0.001). (E-F) Change from baseline in dermatologist assessment score of skin redness. (G-H) Change from baseline in erythema index measured by Mexameter® MX 18. (* *P*-value < 0.05 and *** *P*-value < 0.001 versus baseline, ** *P*-value < 0.01 between emulsion and water control). (I) Representative images taken by Tivi700 of subject at T0, TIMM, T1H and T2H.

Jul.13, 2026

Dear Reviewers,

Re: Manuscript ID: NCOMMS-26-005126B

Title: “Supramolecular Janus Eutectic Engineered from Panthenol and Bisabolol Overcomes Transdermal Delivery Paradoxes”

We would like to express our sincere gratitude to the editor and all reviewers for their positive and constructive comments on our revised manuscript. We are delighted that the reviewers have found our work acceptable for publication in *Nature Communications* pending minor revisions.

We have carefully addressed each of the remaining points raised by the reviewers. All changes have been highlighted in the revised manuscript using tracked changes (or: using red highlighting) for easy identification.

The reviewer’s comments are presented in ***bold italic***, followed by our responses in plain text.

Thank you again for your consideration of our manuscript.

Sincerely,

Jiaheng ZHANG

Sauvage Laboratory for Smart Materials, School of Materials Science and Engineering, Harbin Institute of Technology (Shenzhen), Shenzhen 518055, China.

E-mail: zhangjiaheng@hit.edu.cn

RESPONSES TO REVIEWER #1

The authors have properly addressed all the questions raised by reviewers. It could be accepted for publication.

Response: We sincerely thank the reviewer for their time, expertise, and constructive feedback throughout the review process. We are very grateful for the positive assessment of our revisions.

RESPONSES TO REVIEWER #2

The manuscript has been substantially improved following revision. The authors have adequately addressed all major concerns raised during the review process, resulting in a clearer and more robust study.

My only remaining reservation concerns the presentation of almost all figures. Several composite figures contain up to nine subpanels, making individual images difficult to examine without significant zooming. While this is manageable in the digital version, some details may become difficult or impossible to interpret in a standard A4 printed format. The authors may wish to consider enlarging key panels or redistributing the figures to improve readability.

Response: We sincerely thank the reviewer for this constructive and practical suggestion. We completely agree that the readability of composite figures - especially those containing up to nine subpanels - could be compromised in printed format.

In response, we have thoroughly reorganized and redesigned all unclearly composite figures to improve clarity. Specifically:

- ✧ Figures that originally contained nine subpanels have been split into two separate figures.
- ✧ Key panels have been enlarged and re-arranged to highlight the most critical data.
- ✧ Font sizes, axis labels, and scale bars have been increased to ensure legibility even in A4 printed format.

We believe these revisions have substantially improved the clarity and accessibility of all figures.

RESPONSES TO REVIEWER #3

The revised manuscript is improved and the additional pilot clinical study answers questions regarding the control test material used to show superiority. There seems to be some

improvement from their system relative to water and the "mixture" control. However, questions remain regarding the generalization of the results, and per the reviewer's response, the innovation of the work to justify publication in this journal. The authos state: "This principle has already been most maturely applied in pharmaceutical cocrystals, which share the same conceptual basis. However, a major current challenge is the lack of suitable characterization methods to verify the impact of complex formulation matrices on supramolecular structure. To address this, we have provided a comparative illustration of the two formulations in terms of efficacy and soothing performance, and we hope this adequately clarifies the issue."
From this response the authors state that the work addresses a characterization problem or challenge. How is this consistent with the narrative in the manuscript regarding the rationale for the janus system? To support generalization of a concept, they should provide examples using systems with other componens, where the results also apply. If the approach is not generalizable, those statements should be removed, amended, or the authors should provide evidence to support their conclusion.

Response: We thank the reviewer for this incisive comment. We sincerely apologize for not clearly clarifying the description of pharmaceutical cocrystals in our previous response. Our intention was to convey that the supramolecular complex form offers greater advantages, rather than to address characterization challenges. We apologize again for the ambiguity. We appreciate the reviewer’s suggestion to clarify our position.

In addition, regarding the general applicability of the Janus system, there are currently no further case reports, and our research data in other areas have not yet been published. Upon careful consideration of the reviewer’s concern, we agree that the current dataset does not yet support a claim of generalizability across different component systems. As the reviewer rightly points out, demonstrating generalization would require additional examples using systems with other building blocks, which is beyond the scope of the present study. We have therefore revised the manuscript to remove or amend all statements that imply broad generalizability of the janus system concept, as indicated on lines 111–116 now reads:

This work provides a proof-of-concept for "carrier-free" delivery systems and offers a promising approach for reconstructing bioactive natural molecules through supramolecular engineering. By elucidating a previously underexplored aspect of the interaction between active ingredients and delivery systems, this work provides a practical design strategy for advancing dermatological therapeutics.
